# Impacts of fungal entomopathogens on survival and immune responses of *Aedes albopictus* and *Culex pipiens* mosquitoes in the context of native *Wolbachia* infections

**DOI:** 10.1371/journal.pntd.0009984

**Published:** 2021-11-29

**Authors:** Jose L. Ramirez, Molly K. Schumacher, Geoff Ower, Debra E. Palmquist, Steven A. Juliano

**Affiliations:** 1 USDA, Agricultural Research Service, National Center for Agricultural Utilization Research, Crop Bioprotection Research Unit, Peoria, Illinois, United States of America; 2 School of Biological Sciences, Illinois State University, Normal, Illinois, United States of America; 3 USDA, Agricultural Research Service, Midwest Area, Peoria, Illinois, United States of America; University of Cambridge, UNITED KINGDOM

## Abstract

Microbial control of mosquitoes via the use of symbiotic or pathogenic microbes, such as *Wolbachia* and entomopathogenic fungi, are promising alternatives to synthetic insecticides to tackle the rapid increase in insecticide resistance and vector-borne disease outbreaks. This study evaluated the susceptibility and host responses of two important mosquito vectors, *Ae*. *albopictus* and *Cx*. *pipiens*, that naturally carry *Wolbachia*, to infections by entomopathogenic fungi. Our study indicated that while *Wolbachia* presence did not provide a protective advantage against entomopathogenic fungal infection, it nevertheless influenced the bacterial / fungal load and the expression of select anti-microbial effectors and phenoloxidase cascade genes in mosquitoes. Furthermore, although host responses from *Ae*. *albopictus* and *Cx*. *pipiens* were mostly similar, we observed contrasting phenotypes with regards to susceptibility and immune responses to fungal entomopathogenic infection in these two mosquitoes. This study provides new insights into the intricate multipartite interaction between the mosquito host, its native symbiont and pathogenic microbes that might be employed to control mosquito populations.

## Author summary

Control of mosquitoes via the use of microbes is a promising alternative to synthetic insecticides and a potential solution to tackle the rapid evolution of insecticide resistance in mosquitoes. Recently, a parasitic microbe named *Wolbachia* has been found to render the mosquito resistant to virus infections and it is currently showing great promise in reducing dengue cases on tests conducted in the field. On the other side of the symbiotic spectrum, we have entomopathogenic fungi, who have evolved to naturally infect and kill insects, and offer a unique potential to control mosquito populations. In this study, we examined the effect that native *Wolbachia* can have on the mosquito susceptibility to fungal entomopathogens. Our findings show that while *Wolbachia* does not affect the action of entomopathogenic fungi on mosquitoes, it does influence the expression of important mosquito immune genes, suggesting that *Wolbachia* has a closer interaction with the mosquito response to microbial infections than previously reported. Furthermore, our study provides new records on the susceptibility of two important mosquito vectors in the USA (*Aedes albopictus* and *Culex pipiens*), with *Cx*. *pipiens* showing significant resistance to the action of one fungal entomopathogen tested. This article informs on the mosquito susceptibility and interaction with other microbes that will aid in the selection of fungal entomopathogens to control mosquitoes, especially those that carry native microbes such as *Wolbachia*.

## Introduction

Despite concerted efforts to control vector-borne diseases, outbreaks around the world continue to increase in frequency and intensity [1–4]. In the absence of effective therapeutic drugs against vector borne pathogens, vector control remains the most important component of public health programs around the world [5]. However, the effectiveness of mosquito control, primarily based on the use of chemical pesticides, has been impacted by the rapid evolution of insecticide resistance [6–8]. Microbial and symbiotic control, using symbiotic or entomopathogenic microbes that kill or render the mosquito host less competent in transmitting pathogens, offer an alternative to tackle this increasingly important public health problem [5,9–12].

In this respect, the endosymbiotic α-proteobacterium *Wolbachia* is currently being adopted as a novel strategy to curb the transmission of arboviruses by mosquitoes [13–15]. For instance, transinfection of *Wolbachia w*Mel strain from *Drosophila melanogaster* or *w*AlbB from *Ae*. *albopictus* into *Aedes aegypti*, impaired the mosquito’s ability to transmit dengue [9,16]. Although the main mechanism of *Wolbachia*-derived viral suppression is not clearly established [17,18], changes in the mosquito immune system [19, 20], *Wolbachia*-virus competition and *Wolbachia* modulation of the mosquito methylation patterns have been suggested as potential mechanisms of viral suppression [17,21–24]. In addition to interfering with the replication and transmission of several arboviruses [9], *Wolbachia* has been shown to reduce filarial [25] and *Plasmodium* infection in mosquitoes [9,26] and to interact with the host native microbiota [27], resulting in increased host fitness [28,29]. Furthermore, at least one study reported a *Wolbachia*-mediated protection in *Drosophila melanogaster* fruit fly against a *B*. *bassiana* fungal strain [30]. Hence, a plethora of research provides evidence that *Wolbachia* can influence the host susceptibility to pathogens [28]. In terms of natural *Wolbachia* infections, most *Aedes albopictus* and *Culex pipiens* mosquitoes are infected with *Wolbachia*, however each strain can have different characteristics or interactions with their mosquito hosts [31].

The use of entomopathogenic fungi to control mosquitoes is another strategy that is being considered to suppress mosquito populations [32,33]. Entomopathogenic fungi have the ability to infect its insect host on contact, quickly developing an infection peg and producing chitinases and proteinases that allow them to penetrate the insect cuticle [34,35]. Once inside the insect hemocoel, the fungal entomopathogen multiplies as single cell blastospores, disseminating throughout the mosquito body, up taking nutrients and eventually leading to host death [35]. Differences in host susceptibility to fungal entomopathogens are thought to be due to variations in fungal strain virulence and to host-specific antifungal responses [36,37]

In terms of host responses during multipartite interactions, the mosquito immune system is at the interface of symbiotic and pathogenic interactions with the mosquito host [38,39]. While *Wolbachia* modulation of the mosquito immune system is recognized in heterologous systems of *Wolbachia* transinfection [20,40], it is less clear what effect native *Wolbachia* infections might have on their host. Mosquito responses to *Wolbachia* transinfections, following the artificial transfer from other mosquito species or from other insects, indicates induction of several components of the mosquito immune defense. For instance, transinfections with *w*AlbB in the mosquito *Ae*. *aegypti* led to the activation of the immune signaling pathways Toll and Imd and were thought to protect the mosquito against bacteria and fungi [20]. In addition, transinfections with *w*MelPop and *w*Mel strains of *Wolbachia* led to the upregulation of the melanization cascade. Lastly *Wolbachia* transinfections have been found to alter the host microbial flora [41].

Similar host response profiles have been observed during pathogenic interactions. For instance, during fungal entomopathogen-mosquito-microbiota interactions, fungal entomopathogens induce a range of mosquito responses, ranging from activation of canonical immune signaling pathways, antimicrobial effectors, oxidative stress and the melanization cascade [35,37,42]. Interestingly, entomopathogenic fungi also interact with the mosquito gut microbiota, creating an environment that leads to dysbiosis of the mosquito gut [36,43]. Furthermore, our studies with the mosquito *Ae*. *aegypti* have shown that the mosquito infection-responsive repertoire tends to display a level of compartmentalization with tissue-specific expression that is fungal strain-specific [36].

In this study we explored the effects of fungal entomopathogenic infections on the survival and immune responses of two important mosquito vectors, *Ae*. *albopictus* and *Cx*. *pipiens* in the context of native *Wolbachia* infections. Our study shows that while *Wolbachia* did not affect the susceptibility of either mosquito species to entomopathogenic fungi, it had significant influence on the microbial load and mosquito transcriptional responses to fungal infection. Such responses, though vastly modulated by fungal infection, were also affected by the presence of native *Wolbachia*, and in some cases, effects of fungal infection and *Wolbachia* interacted. This study expands our knowledge of fungal entomopathogenic susceptibility in two important mosquito species and provides a snapshot of the molecular interactions of natural *Wolbachia* infections with mosquitoes during a pathogenic infection process.

## Materials and methods

### Mosquito rearing

*Aedes albopictus* eggs were provided by collaborators at Tyson Research Center, Washington University, Eureka, MO and reared at the National Center for Agricultural Utilization Research in Peoria, IL. All eggs, larvae, pupae, and adults were housed at 28°C with 14:10 light:dark photoperiod. Eggs were allowed to hatch for 72 hours in a 12 x 10 x 3 photo developer tray with 1L of DI water and maintained on a mixture of rabbit food, fish food, and liver powder. Adults were provided with 10% sucrose solution and were provided with a blood meal at 3–6 days post emergence using an artificial membrane feeder and bovine blood (Hemostat). *Culex pipiens* were collected in Normal, IL and reared at Illinois State University, Normal, IL. Adults of the original *Wolbachia*(+) *Cx*. *pipiens* colony were housed in an insectary at ~25°C with a 16:8 light:dark photoperiod with a 2-hour dawn/dusk phase. To avoid cross-contamination between colonies with *Wolbachia*, adults of the *Wolbachia*-free *Cx*. *pipiens* colony were housed in a separate walk-in environmental room at 25°C, with a 14:10 light:dark photoperiod. Adults of both colonies were provided with 20% sucrose solution and blood fed during the dark phase from anesthetized laboratory mice (IACUC protocol #842043) placed on the screen top of the cage. Custom-made mesh magnetic cages were placed over feeding females after they settled on the mice to limit each mouse to fewer than 25 bites. To encourage synchronous egg laying and to provide ample water surface area, 5 days post-blood feeding 7.5 L buckets containing white oak leaf infusion were placed in the cages. Egg rafts from both colonies were removed the next day and placed in separate beakers with white oak leaf infusion. The hatched larvae were counted into cohorts of 500 larvae, placed into 3 L of 2 g/L white oak infusion in 7.5 L buckets, and transported to the National Center for Agricultural Utilization Research in Peoria, IL, where experimental larvae were reared to adulthood at 28°C with a 14:10 light:dark photoperiod. The water level was maintained at 3 L by adding DI water as needed. Larvae were given bovine liver powder daily and 3 g of timothy hay was added to the buckets after they reached 3rd instar. Experimental adults were provided with 10% sucrose solution, and adult females from both species entered experiments when they reached 3–5 days old.

### Entomopathogenic fungal strains and infection bioassays

To evaluate the effect of natural *Wolbachia* infection in the mosquito susceptibility to fungal entomopathogens we used two entomopathogenic fungal strains: *Beauveria bassiana* (MBC076) and *Beauveria brongniartii* (MBC397). Infection bioassays were conducted as previously described [36]. Briefly, fungal cultures were grown on ¼ strength Sabouraud dextrose agar yeast extract (SDAY) medium and conidia oil formulations were prepared from 15-day old cultures using soybean oil as a carrier. Following homogenization, the mixture was filtered through cheese cloth and conidia counted using an improved Neubauer hemocytometer. The suspension was adjusted to a conidial concentration of 1 x 10^8^ conidia/ml. Topical exposure was conducted by depositing 50.6 nl of the conidial suspension (equivalent to 50,600 conidia/mosquito) on the coxal region of cold-anesthetized mosquitoes via a Nanoject III micropipet. Mosquitoes from the control group were exposed to the same treatment and exposed to the same volume of soybean oil devoid of any fungal conidia. At least three independent experiments were conducted for survival assays and for analysis of gene expression. Each experiment was conducted with fresh batches of mosquitoes and fungal suspensions. Treated mosquitoes were transferred and maintained in insect-cup cages under standard insectary conditions and provided with 10% sucrose solution. All experimental mosquitoes were maintained solely on sucrose solution and at no time were allowed to blood-feed. Mosquito survival was monitored daily, and mosquito cadavers removed from the cage. Survival curves from each treatment were analyzed via Kaplan-Meier estimator with median survival time differences between each treatment compared via Log-rank test (GraphPad Prism9.0). The lethal time to 50% mortality (LT_50_) values were calculated by probit analysis.

### *Wolbachia* clearance

*Wolbachia* clearance from both mosquito species was conducted via tetracycline treatment of the adult mosquitoes as previously specified [44]. Briefly, adult mosquitoes were separated at the time of emergence into control (*Wolbachia* (+)), and tetracycline-treated cohorts (*Wolbachia* (-)), and provided with either sugar alone or sugar meals laden with 1.25 mg/ml of tetracycline respectively. All sugar meals were replaced every other day and following blood meals. *Wolbachia* clearance was verified via qPCR using DNA extracted (DNeasy Blood and Tissue Kit, QIAGEN) and *Wolbachia*-specific primers (S1 Table) following the methodology from [45] from subsamples of 5–10 adult males and females randomly collected from each treated group. Five and three generations of tetracycline treatment were necessary to completely clear *Wolbachia* from *Ae*. *albopictus* and *Cx*. *pipiens* mosquitoes respectively. To conduct bioassays and once clearance of *Wolbachia* was confirmed, eggs from both treatment groups were hatched and larvae and adult mosquitoes were reared in the absence of tetracycline for the remainder of the experimental procedures. *Wolbachia* strain identification via PCR in *Aedes albopictus* mosquitoes determined that these mosquitoes were superinfected with *w*AlbA and *w*AlbB strains, while *Cx*. *pipiens* mosquitoes carried the *w*Pip strain. All qPCR screening assays included samples randomly picked from the W+ cohorts to serve as positive controls for DNA extraction and qPCR-based *Wolbachia* detection.

### Gene expression analyses

Gene expression analyses was conducted on pools of 5 mosquitoes collected at 6d post-infection (PI). The time point was selected based on our previous assays with the mosquito *Ae*. *aegypti* and it corresponds to the late stages of infection [36]. RNA from whole body homogenates were extracted using TRizol (Invitrogen) following the manufacturer’s instructions. RNA concentration and purity were assessed via Nanodrop (Thermo Scientific). RNA samples were normalized to 1μg and then used in cDNA synthesis using the QuantiTec reverse transcription kit with DNA Wipeout (Qiagen). Gene expression analysis was conducted using the PowerUp SYBR green Master mix qPCR kit (Qiagen) and gene-specific primers (S1 Table) in a 10 μl reaction using one microliter of cDNA. Primers used in this study were those available in the literature or designed for this study based on orthology via VectorBase, using the structural annotation version AaloF1.2 for *Ae*. *albopictus* and CpipJ2.5 for a representative of the *Cx*. *pipiens* complex, *Cx*. *quinquefasciatus*. VectorBase uses the OrthoMCL algorithm for homolog predictions [46]. The resulting protein gene sequences from the OrthoMCL’s ortholog groups were used with Clustal Omega to create a phylogenetic tree of the alignment for *Ae*. *aegypti*, *Ae*. *albopictus*, *Cx*. *quinquefasciatus* and *Anopheles gambiae* gene targets (See Sup document 1). We employed the RT-qPCR cycling conditions recommended for the master mix and it consisted of holding at 95.0°C for 10min and 40 cycles of 15 s at 95.0°C and 1min at 60°C. Melt curve analysis was included at the end of each qPCR run. Each sample was assayed in duplicate (technical replicates) and the reproducibility of the results evaluated with at least 3 independent experiments (2 to 4 biological replicates per experiment) conducted with fresh batches of mosquitoes and fungal suspensions. To normalize cDNA samples, we evaluated the expression of *Rps7* and *Rps3* for *Ae*. *albopictus* and *Cx*. *pipiens*, respectively. The RT-qPCR assays were conducted on an Applied Biosystems QuantStudio 6 Flex Real-time PCR system (ThermoFisher Scientific). Transcript abundance was evaluated post-run using the ΔΔCt method [47] with data from three independent experiments pulled in the analysis.

### Phenoloxidase assays

Phenoloxidase activity (PO) as conducted as previously reported [36]. Briefly, mosquitoes were collected at six days post-infection, pairs of two cold-anesthetized mosquitoes from each factorial treatment were placed in a 2 mL tube with 50 μL of chilled 1x PBS and a 2.4 mm bead. Samples were macerated in a TissueLyserII (QIAGEN) for 30s at 30Hz. The homogenized samples were immediately snap-frozen in liquid nitrogen to prevent enzyme catalyzation, thawed on ice and centrifuged at 10000 rpm for 5 min at 4C. Here, 35 μL of the supernatant were transferred to a 1.5 mL centrifuge tube, frozen in liquid nitrogen and stored at -80°C until ready for active PO assay. All reactions were prepared on ice except when noted. In duplication, 15 μL of samples were added to a flat-bottomed 96-well plate well that also contained 140 μL chilled distilled water and 20 μL cold PBS. Two wells were filled with an additional 15 μL and no sample to serve as a blank for the non-enzymatic production of dopaquinone. To each well was added 20 μL of L-Dopa solution (4 mg per mL H2O; 3,4 dihydroxy-L-phenylalanine) and read with a spectrophotometer (Multiskan GO, Thermal Scientific). Change in absorbance was measured at 490nm for 30 min at 30°C and measured every 15s. Enzymatic activity was calculated as the slope (Vmax) of the reaction curve during its linear phase. At least three independent experiments were conducted with 10 samples per treatment and per experiments employed.

### Statistical analyses

For qPCR data and PO data, outliers were identified via GraphPad statistical software and removed from the analysis. A total of 25 out of 1407 data points were identified as outliers in *Ae*. *albopictus* and 45 out of 1393 data points in *Cx*. *pipiens*. A 2-way ANOVA was performed within gene targets for each species using a generalized linear model (PROC GLIMMIX, SAS 9.4) with a Gamma distribution of error and a log link function. The SAS ILINK option was used to express least squares means and confidence intervals on the original scale. Significant effects were further analyzed by pairwise comparisons of the gamma distributed estimates for the main effects of fungal treatment (F), with 3 levels (*B*. *bassiana*, *B*. *brongniartii*, uninfected control) *Wolbachia* treatment (W) with 2 levels (*W+*, *W-*) and their interaction (F*W) with a Tukey adjustment for multiple comparisons. Graphical representation of the data was done using Graph-Pad Prism 9 (GraphPad). Analyses for fungal effects on *Wolbachia* load were done using only the *Wolbachia* infected groups in a one-way ANOVA, with the same 3 levels (*B*. *bassiana*, *B*. *brongniartii*, uninfected control), using a generalized linear model (PROC GLIMMIX, SAS 9.4) with a Gamma distribution of error and a log link function. Pairwise comparisons among means also used a Tukey adjustment.

## Results

### Clearance of natural Wolbachia does not affect mosquito susceptibility to fungal entomopathogens

**Fig 1:**
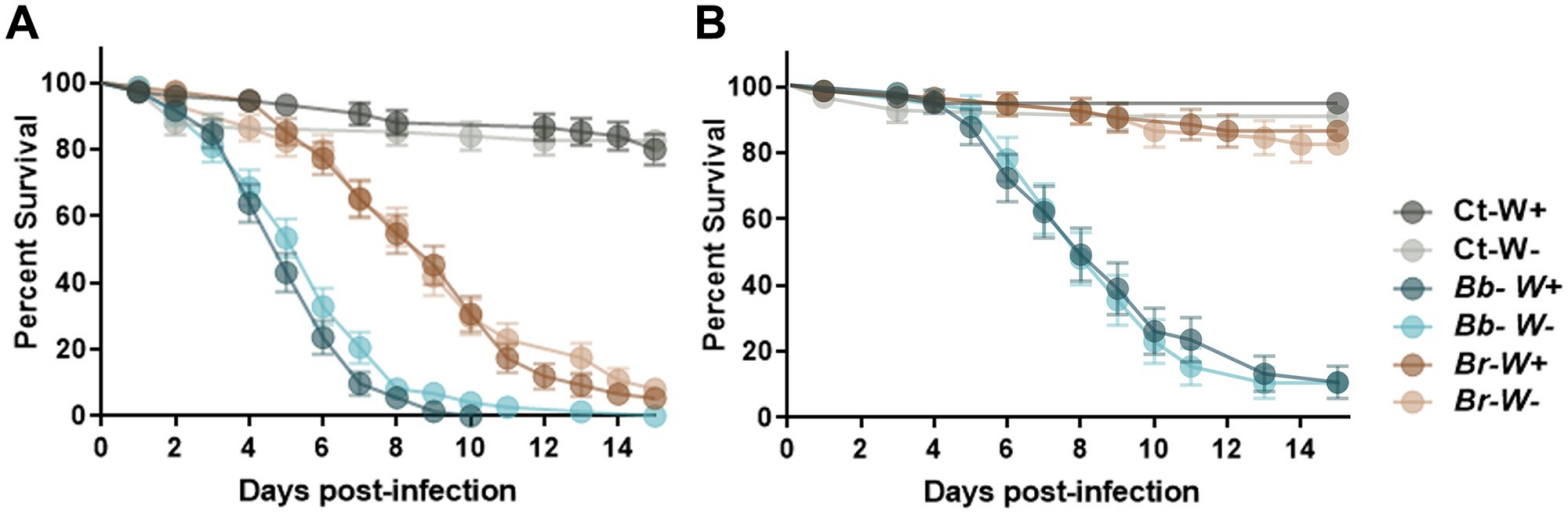
Survival curves of *Wolbachia*-infected (W^+^) and *Wolbachia*-free (W^-^). *Ae*. *albopictus* (A) and *Cx*. *pipiens* (B) mosquitoes following challenge with either *Beauveria bassiana* (Bb) or *Beauveria brongniartii* (Br) fungal entomopathogens. Ct = control group exposed to soy oil carrier without fungal spores. Survival graphs represents five independent experimental replicates (total n = 75 individuals per treatment) for *Ae*. *albopictus* and four independent experimental replicates (total n = 40 individuals per treatment) for *Cx*. *pipiens*. Data was analyzed with Log-rank Mantel-Cox test.

To evaluate whether the presence of *Wolbachia* affected the susceptibility of mosquitoes to fungal entomopathogens, *Wolbachia*-infected (*W*^*+*^) and *Wolbachia*-free (*W*^*-*^) mosquitoes maintained solely with sucrose solutions, were infected with either *B*. *bassiana* or *B*. *brongniartii* entomopathogenic fungi. Overall, mosquito survival post fungal infection differed significantly with each fungal strain and mosquito host. However, mosquito survival post-fungal infection did not differ between *W*^*+*^ and *W*^*-*^ mosquitoes. This was observed in both *Ae*. *albopictus* infected with *B*. *bassiana* (log-rank Mantel-Cox test, χ^2^: 2.8, *P* = 0.0933) or *B*. *brongniartii* (log-rank Mantel-Cox test, χ^2^: 0.34, *P* = 0.5608), and in *Cx*. *pipiens* infected with either *B*. *bassiana* (log-rank Mantel-Cox test, χ^2^: 0.03, *P* = 0.8572) or *B*. *brongniartii* (log-rank Mantel-Cox test, χ^2^: 0.24, *P* = 0.6231) (Fig 1).

Likewise, Probit analysis indicated no difference in LT_50_ values between *W*+ and *W*- mosquitoes when infected with either of the two entomopathogenic fungi. However, we observed a difference in susceptibility in these two mosquito species, with *Ae*. *albopictus* showing greater susceptibility to *B*. *bassiana* (LT_50_: 4.5 days; 95% CI: 3.6–5.5 days) and to *B*. *brongniartii* (LT_50_: 8.35 days; 95% CI: 4.8–11.9 days) than *Cx pipiens*. In fact, *Cx*. *pipiens* mosquitoes were less susceptible to *B*. *bassiana* spores (LT_50_: 7.9 days; 95%CI: 5.5–10.3 days) and highly resistant to *B brongniartii* infection. The LT_50_ for *B*. *brongniartii-*infected *Cx*. *pipiens* could not be determined due to mosquito survival in this cohort exceeding 50% at the end of the experiment.

We present our qPCR analyses in sets of genes with related functions. These sets are: Immune signaling genes (*Rel 1*, *Rel 2*, *PGRP-LC*, *PGRP-S1*); antimicrobial effector genes (*CecA*, *DefC*, *LysE*, *Tep22*, *LysC*); oxidative stress response genes (*Duox*, *DuoxA*); antioxidant defense genes (Catalase, *CuZnSOD*, *GPX*, *OXR*, *GST*); pro-phenoloxidase genes (*PPO1-PPO9*); and genes that indicate bacterial, fungal, and *Wolbachia* loads (*16srRNA*, *18srRNA*, *Wolbachia wsp*, respectively). We also present a similar analysis of phenoloxidase activity level.

### Expression of immune signaling pathways and antimicrobial effectors are more affected by fungal infection than Wolbachia infection status

**Fig 2:**
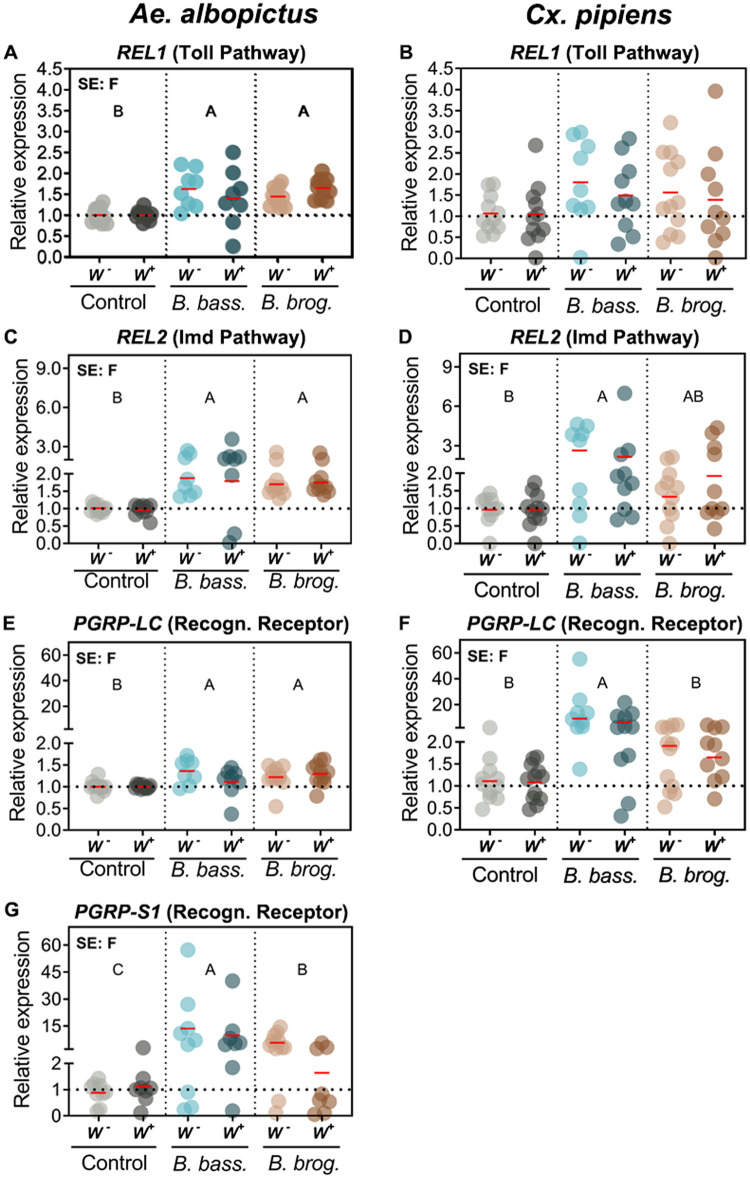
Gene expression of mosquito immune signaling pathway components under the context of natural *Wolbachia* and fungal entomopathogenic infections. Significant effects (SE) indicate whether the independent factors: Fungal entomopathogen (F), *Wolbachia* presence (W) or their interaction (F*W) were statistically significant. The red horizontal line indicates LS-means from eight to eleven biological replicates per treatment, originating from at least three independent experiments. Groups sharing the same letter are not significantly different at p<.05 based on differences of least-squares means. Uppercase letters refer to statistically significant fungal effects. W^-^, *Wolbachia*-free; W^+^, *Wolbachia*-infected; *B*. *bass*., *B*. *bassiana*; *B*. *brog*., *B*. *brongniartii*. See Table 1 for complete statistics from the Two-Way ANOVA.

To further assess the interaction between *Wolbachia* infection status and fungal entomopathogenic infection, we evaluated the expression of key anti-fungal immune markers that have been shown to be important in the *Ae*. *aegypti* immune response to these same entomopathogenic fungi. Our analysis showed no significant interactive effect of fungal strain and *Wolbachia* presence, and no significant main effect of *Wolbachia*, on the immune signaling pathways of both *Ae*. *albopictus* and *Cx*. *pipiens* mosquitoes (Fig 2 and Table 1). However, there was a significant effect of fungal infection on pathogen recognition receptors *PGRP-LC* (*p* < 0.0013), *PGRP-S1* (*p* < 0.0001), and transcription factors *REL1* (Toll Pathway) (*p* < 0.0001) and *REL2* (Imd pathway) (*p* < 0.0001) in the mosquito *Ae*. *albopictus* (Table 1). This significant increase in expression was observed independent of fungal strain, as we observed high significant induction when *Ae*. *albopictus* mosquitoes were infected with either *B*. *bassiana* or *B*. *brongniartii* compared to the uninfected controls (Fig 2).

**Table 1:** Interactive effects of 2-way ANOVA for microbial load, immune signaling pathways and AMPs (qPCR Type III Fixed effects). Arrows indicate up or down-regulation of gene expression during fungal or *Wolbachia* infection.

	Target	Effect	*Ae*. *albopictus*			*Cx*. *pipiens*		
			Num/Den DF	F Value	Pr>F	Num/Den DF	F Value	Pr>F
**Imm. Sign.** **Pathways**	**PGRP-LC**	F	2/54	**↑**7.57	***0*.*0013***	2/57	**↑**45.38	***<0*.*0001***
		W	1/54	0.77	0.3831	1/57	1.21	0.2763
		F*W	2/54	1.96	0.1509	2/57	0.38	0.6844
	**PGRP-S1**	F	2/47	**↑**23.56	***<0*.*0001***			
		W	1/47	2.31	0.1355			
		F*W	2/47	2.31	0.1109			
	**Rel1**	F	2/54	**↑**18.72	***<0*.*0001***	2/57	1.94	0.1525
		W	1/54	0.01	0.9078	1/57	0.31	0.5782
		F*W	2/54	1.36	0.2662	2/57	0.07	0.936
	**Rel2**	F	2/51	**↑**12.33	***<0*.*0001***	2/55	**↑**4.62	***0*.*014***
		W	1/51	0.03	0.8641	1/55	0.05	0.8214
		F*W	2/51	0.04	0.9565	2/55	0.47	0.6293
**Antimicrobial** **Effectors**	**CecA**	F	2/54	**↑**36.64	***<0*.*0001***	2/55	**↑**9.2	***0*.*0004***
		W	1/54	2.47	0.1222	1/55	**↓**18.96	***<0*.*0001***
		F*W	2/54	0.55	0.5782	2/55	3.04	0.0557
	**DefC**	F	2/52	**↑**55.37	***0*.*0001***	2/50	**↑**16.84	***<0*.*0001***
		W	1/52	0.99	0.3248	1/50	0.66	0.4199
		F*W	2/52	1.33	0.2735	2/50	4.68	***0*.*0137***
	**Lys**	F	2/53	**↑**43.29	***<0*.*0001***	2/55	**↑**5.15	***0*.*0089***
		W	1/53	0.23	0.6354	1/55	0.4	0.5275
		F*W	2/53	0.38	0.6862	2/55	1.9	0.1588
	**Tep22**	F	2/52	0.59	0.5594	2/56	**↑**7.32	***0*.*0015***
		W	1/52	**↑**12.16	***0*.*001***	1/56	**↓**4.74	***0*.*0337***
		F*W	2/52	3.73	***0*.*0305***	2/56	0.22	0.806
**Oxidative Stress**	**Duox**	F	2/53	**↑**4.21	***0*.*02***	2/59	0.03	0.973
		W	1/53	2.59	0.1135	1/59	0.47	0.4955
		F*W	2/53	2.49	0.0922	2/59	0.33	0.7234
	**DuoxA**	F	2/55	**↑**3.39	***0*.*0408***			
		W	1/55	1.31	0.2565			
		F*W	2/55	0.05	0.9471			
**Antioxidant Defense**	**Catalase**	F	2/51	0.86	0.4283	2/56	**↑**6.56	***0*.*0028***
		W	1/51	**↓**8.29	***0*.*0058***	1/56	**↑**15.46	***0*.*0002***
		F*W	2/51	4.84	***0*.*0119***	2/56	3.82	***0*.*0278***
	**GPX**	F	2/55	**↑**10.21	***0*.*0002***	2/55	0.28	0.758
		W	1/55	0.18	0.6705	1/55	0.5	0.4816
		F*W	2/55	0.41	0.6662	2/55	1.47	0.2389

In contrast, our analysis of *Cx*. *pipiens* mosquitoes indicated a significant effect of fungal infection on *PGRP-LC* (*p* < 0.0001) and *REL2* expression (*p* < 0.014); but not on *REL1* (Table 1). This induction however was observed only when *Cx*. *pipiens* mosquitoes were infected with *B*. *bassiana*. Although *B*. *brongniartii*-infected *Cx pipiens* mosquitoes presented an increase in expression, it was not statistically significant (Fig 2). We did not assess *Cx pipiens PGRP-S1*, as repeated attempts designing a working primer set did not produce a unique PCR product and was not included in our qPCR assessment.

**Fig 3:**
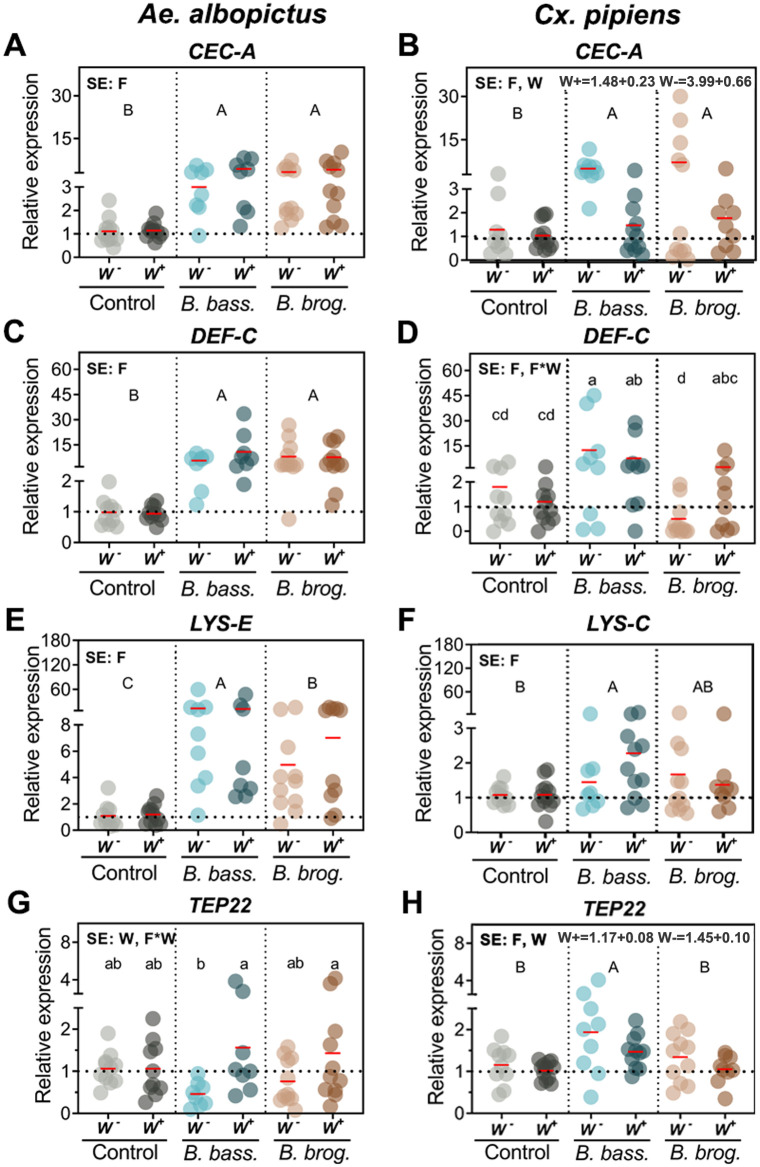
Gene expression of anti-microbial effectors as a result of natural *Wolbachia* and fungal entomopathogenic infections. Significant effects (SE) indicate whether the independent factors: Fungal entomopathogen (F), *Wolbachia* presence (W) or their interaction (F*W) were statistically significant. Lowercase letters indicate interactive effects (F*W), uppercase letters refer to fungal effects and any *Wolbachia* effect is represented by their mean and standard deviation on the upper right corner of the graph. The red horizontal line indicates LS-means from eight to eleven biological replicates per treatment, originating from at least three independent experiments. Groups sharing the same letter are not significantly different at p<0.05 based on differences of least-squares means. W^-^, *Wolbachia*-free; W^+^, *Wolbachia*-infected; *B*. *bass*., *B*. *bassiana*; *B*. *brog*., *B*. *brongniartii*. *CecA*, Cecropin A; *Def-C*, Defensin C; *Lys-E*, Lysozyme E; *Lys-C*, Lysozyme C; *TEP22*, Thioester-containing protein 22. See Table 1 for complete statistics from the Two-Way ANOVA.

We further evaluated the gene expression of antimicrobial effectors whose orthologs in *Ae*. *aegypti* have been shown to be elicited upon fungal infection. We found significant interactive effects of fungal infection and *Wolbachia* only for *TEP22* (*p* < 0.0305) for *Ae*. *albopictus*, and only for *DEFC* for *Cx*. *pipiens* (*p* < 0.0137) (Table 1). There were significant main effects of fungal infection on the expression of *CECA* (*p* < 0.0001), *DEFC* (*p* = 0.0001), *LYSE* (*p* < 0.0001) in *Ae*. *albopictus* and on *CECA* (*p* < 0.0004), *DEFC* (*p* < 0.0001), *LYSC* (*p* = 0.0089) and *TEP22* (*p* = 0.0015) in *Cx*. *pipiens* (Table 1). Both entomopathogenic fungi, *B*. *bassiana* and *B*. *brongniartii*, significantly induced the expression of *CECA* in both mosquitoes, and *Wolbachia* presence yielded significantly lower *CECA* expression (*p* < 0.0001) in *Cx*. *pipiens* (Fig 3B).

Defensin expression had a profile similar to cecropin, with fungal infection inducing upregulation of *DEFC* in *Ae*. *albopictus* (*p* < 0.0001) and *Cx pipiens* (*p* < 0.0001) mosquitoes. While *DEFC* induction in *Ae*. *albopictus* was significantly upregulated during infection with either fungal entomopathogen (Fig 3C), in *Cx*. *pipiens* the interaction arose because both *W-* and *W+ B*. *bassiana* infected adults yielded mean expressions significantly greater than those for corresponding uninfected controls (Fig 3D), but both *W-* and *W+ B*. *brongniartii* infected adults yielded mean expressions not significantly greater than those for uninfected controls (Fig 3D). Further, *W-* adults had significantly greater *DEFC* expression when infected with *B*. *bassiana* than when infected *B*. *brongniartii*, but the difference between the two fungal species was not significant in *W+* adults (Fig 3D).

The effect of fungal infection on lysozyme expression was highly significant but differed between mosquitoes and with fungal strain. In *Ae*. *albopictus*, *LYSE* expression was significantly higher (*p* < 0.0001) in *B*. *bassiana* and *B*. *brongniartii*-infected mosquitoes compared to the control groups. However, *LYSE* expression was significantly higher in *B*. *bassiana*-infected mosquitoes than in those infected with *B*. *brongniartii* (Fig 3E). In contrast, increased expression of *LYSC* in *Cx*. *pipiens* was only statistically significant for *B*. *bassiana*-infected mosquitoes (*p* = 0.0089) (Fig 3F). We also evaluated the expression of *TEP22*, whose ortholog in *Ae*. *aegypti* functions as a potent anti-fungal effector. For *TEP22* in *Ae*. *albopictus*, mean expression was significantly greater for *W+* than for *W-* adults when infected with *B*. *bassiana*, but this difference was not statistically significant when infected with *B*. *brongniartii* or when uninfected (Fig 3G). Differences in *TEP22* expression between *W*^*-*^ and *W*^*+*^ *Ae*. *albopictus* were similar in direction for both fungus infected groups (Fig 3G), with lower *TEP22* expression in *W*^*-*^ than in *W*^*+*^. *Wolbachia* infection affected *Ae*. *albopictus* and *Cx*. *pipiens TEP22* expression differently, with *TEP22* expression increasing in fungus infected *W*^+^ compared to *W*^*-*^ *Ae*. *albopictus*, but decreasing significantly in *W*^*+*^ compared to *W*^*-*^ *Cx*. *pipiens* regardless of infection (Compare Fig 3G and 3H).

### Fungal infection alters the state of oxidative stress and induces the antioxidant defense in a Wolbachia and fungal strain dependent manner

**Fig 4:**
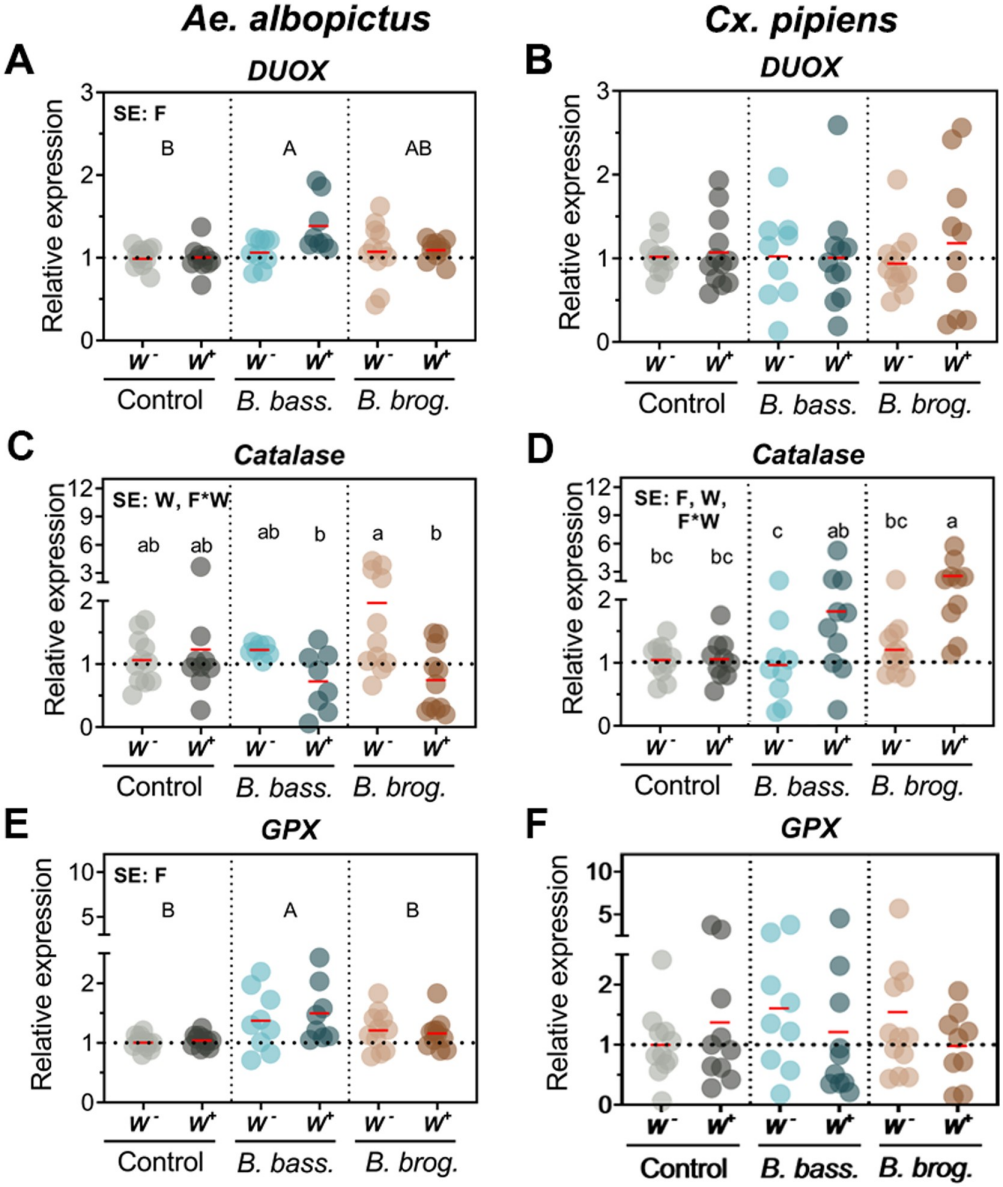
Gene expression of oxidative stress and detoxification genes during natural *Wolbachia* and fungal entomopathogenic infections. Significant effects (SE) indicate whether the independent factors: Fungal entomopathogen (F), *Wolbachia* presence (W) or their interaction (F*W) were statistically significant. Lowercase letters indicate interactive effects (F*W), uppercase letters refer to fungal effects and any *Wolbachia* effect is represented by their mean and standard deviation on the upper right corner of the graph. The red horizontal line indicates LS-means from eight to eleven biological replicates per treatment, originating from at least three independent experiments. Groups sharing the same letter are not significantly different at p<0.05 based on differences of least-squares means. W^-^, *Wolbachia*-free; W^+^, *Wolbachia*-infected; *B*. *bass*., *B*. *bassiana*; *B*. *brog*., *B*. *brongniartii*. See Table 1 for complete statistics from the Two-Way ANOVA.

To understand the implication of the oxidative stress pathway and antioxidant defense system during a fungal entomopathogenic infection and *Wolbachia* presence, we evaluated the expression of several genes involved in oxidative stress and antioxidant defense. Our transcript abundance analysis indicated a significant effect of fungal infection on the expression of dual oxidase genes *DUOX* (*p* = 0.02) (Fig 4A) and *DUOXA* (*p* = 0.0408) (S1 Fig) in *Ae*. *albopictus* mosquitoes. This however was fungal strain dependent, with *Ae*. *albopictus* mosquitoes presenting a significant increase in *DUOX* expression only when challenged with *B*. *bassiana* but not when infected with *B*. *brongniartii* (Fig 4A). In comparison, the expression of a *DUOX* gene in *Cx pipiens* (*DUOX*) did not show any significant change (*p* = 0.973) in transcript abundance with either fungal or *Wolbachia* infection status. Further analysis of genes involved in the antioxidant defense indicated a significant effect of *B*. *bassiana* infection on *GPX* expression (*p* = 0.0002) only in *Ae*. *albopictus* mosquitoes (Fig 4E and Table 1).

We found significant interactions of fungal infection and *Wolbachia* for expression of the catalase gene in both mosquito species (*Ae*. *albopictus*, *p* = 0.0119) (*Cx*. *pipiens*, *p* = 0.0278) (Table 1). The direction and magnitude of catalase gene induction differed between these two mosquitoes. *W*^*-*^ *Ae*. *albopictus* mosquitoes presented greater expression of catalase compared to their *W*^*+*^ counterpart when challenged with fungal infection and that difference was significant in pairwise comparisons for *B*. *brongniartii*, but not for *B*. *bassiana* infection (Fig 4C). In contrast, *W*^*-*^ *Cx*. *pipiens* mosquitoes presented lesser expression of catalase compared to their *W*^*+*^ counterpart when infected with either of the fungal entomopathogens (Fig 4D). This differential response of *W*^*+*^ and *W*^*-*^ mosquitoes was absent in the fungus-free controls (Fig 4C and 4D). We observed no change in expression for antioxidant defense genes *CuZnSOD* and *OXR1* in *Ae*. *albopictus* and GST in *Cx pipiens* mosquitoes (S2 Fig and S2 Table).

### Pro-phenoloxidase genes are differentially elicited as a result of natural Wolbachia and fungal entomopathogenic infections

**Fig 5:**
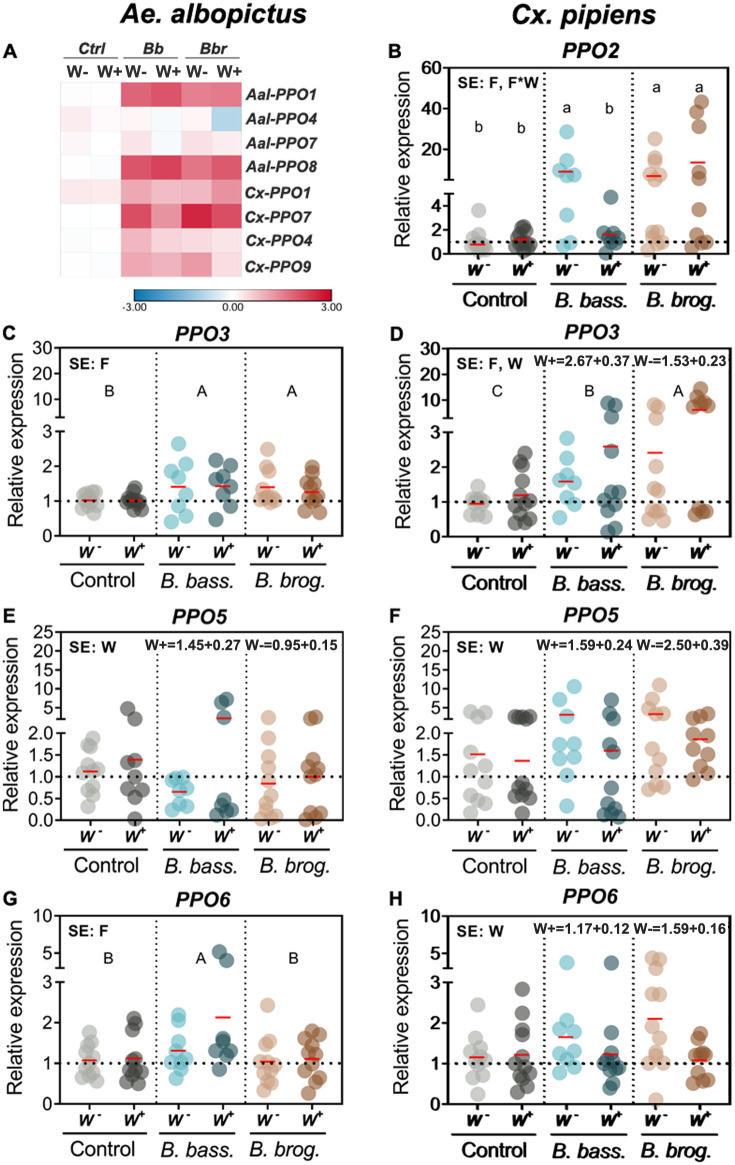
Gene expression of pro-phenoloxidase genes during natural *Wolbachia* and fungal entomopathogenic infections. (A) Heatmap of PPO genes affected only by fungal infection in both *Ae*. *albopictus* and *Cx pipiens*. (B-H) Varying effects of independent factors on the relative expression of PPO genes of both mosquito species. Heatmap represents the log2 LS-mean values, with red color indicating upregulation and blue color downregulation in comparison to the controls. Gene expression Significant effects (SE) indicate whether the independent factors: Fungal entomopathogen (F), *Wolbachia* presence (W) or their interaction (F*W) were statistically significant. Lowercase letters indicate interactive effects (F*W), uppercase letters refer to fungal effects and any *Wolbachia* effect is represented by their mean and standard deviation on the upper right corner of the graph. The red horizontal line indicates LS-means from eight to eleven biological replicates per treatment, originating from at least three independent experiments. Groups sharing the same letter are not significantly different at p<0.05 based on differences of least-squares means. W^-^, *Wolbachia*-free; W^+^, *Wolbachia*-infected; *B*. *bass*., *B*. *bassiana*; *B*. *brog*., *B*. *brongniartii*. *PPO*, pro-phenoloxidase. See Table 2 for complete statistics from the Two-Way ANOVA.

Given the important role that pro-phenoloxidase (PPO) cascade plays in the mosquito response to fungal infection, we assessed PPO genes in these two mosquitoes. Across all PPO genes, *Cx*. *pipiens* expression yielded consistently more effects of fungal infection, *Wolbachia*, and their interaction (Table 2). There was a significant interaction between *Wolbachia* and fungal infection only for *PPO2* expression, and only in *Cx*. *pipiens* mosquitoes (*p* = 0.0005) (Table 2). Fungal infection significantly induced *PPO2* expression (*p* < 0.0001) compared to control for *W*^*-*^ *Cx*. *pipiens* infected with *B*. *bassiana*, and for both *W*^*+*^ and *W*^*-*^ *Cx*. *pipiens* infected with *B*. *brongniartii* (Fig 5B). In contrast, *W*^*+*^ *Cx*. *pipiens* infected with *B*. *bassiana* did not show significant gene induction compared to control (Fig 5B).

**Table 2:** Interactive effects of 2-way ANOVA for PPO expression and phenoloxidase activity (qPCR Type III Fixed effects). Arrows indicate up or down-regulation of gene expression during fungal or *Wolbachia* infection.

	Target	Effect	*Ae*. *albopictus*			*Cx*. *pipiens*		
			Num/Den DF	F Value	Pr>F	Num/Den DF	F Value	Pr>F
**Pro-phenoloxidase**	**PPO1**	F	2/53	**↑**58.07	***<0*.*0001***	2/59	**↑**3.86	***0*.*0265***
		W	1/53	0.21	0.6523	1/59	0.07	0.7967
		F*W	2/53	0.17	0.8444	2/59	0.61	0.5482
	**PPO2**	F				2/54	**↑**33.28	***<0*.*0001***
		W		-		1/54	0.74	0.3949
		F*W				2/54	8.7	***0*.*0005***
	**PPO3**	F	2/53	**↑**5.14	***0*.*0091***	2/56	**↑**13.85	***<0*.*0001***
		W	1/53	0.11	0.7381	1/56	7.28	***0*.*0092***
		F*W	2/53	0.17	0.8455	2/56	1.07	0.3484
	**PPO4**	F	2/54	1.25	0.2949	2/59	**↑**10.22	**0.0002**
		W	1/54	2.32	0.1335	1/59	0.97	0.3276
		F*W	2/54	0.92	0.4033	2/59	0.96	0.3887
	**PPO05**	F	2/52	0.63	0.538	2/58	2.44	0.0961
		W	1/52	**↑**4.27	***0*.*0437***	1/58	**↓**4.23	**0.0442**
		F*W	2/52	1.60	0.2118	2/58	0.66	0.5186
	**PPO6**	F	2/55	**↑**4.98	***0*.*0103***	2/59	1.08	0.3475
		W	1/55	2.75	0.1029	1/59	**↓**4.61	***0*.*0359***
		F*W	2/55	1.26	0.2923	2/59	2.24	0.1155
	**PPO7**	F	2/53	1.39	0.2589	2/54	**↑**15.25	***<0*.*0001***
		W	1/53	2.99	0.0898	1/54	1.32	0.2563
		F*W	2/53	0.54	0.5845	2/54	0.41	0.6638
	**PPO8**	F	2/52	**↑**60.97	***<0*.*0001***			
		W	1/52	0.82	0.3693			
		F*W	2/52	0.55	0.5815			
	**PPO9**	F				2/56	**↑**6.32	***0*.*0034***
		W				1/56	1.92	0.1712
		F*W				2/56	1	0.3726
**Phenoloxidase**	**Phenoloxidase Activity**	F	2/18	1.62	0.2254	2/18	**↑**4.37	***0*.*0284***
		W	1/18	0.25	0.6251	1/18	0.93	0.3475
		F*W	2/18	1.94	0.1719	2/18	0.12	0.8843

The main effect of fungal infection differentially affected the expression of PPO genes in these two mosquitoes. The fungal effect on the expression was significant for four (*PPO1*, *PPO3*, *PPO6*, *PPO8*) out of seven PPO genes in *Ae*. *albopictus*, and six (*PPO1*, *PPO2*, *PPO5*, *PPO7*, *PPO4* and *PPO9*) out of eight PPO genes in *Cx pipiens* (Table 2). The greatest increase in PPO transcript expression (>2.1-fold relative to controls) due to fungal infection was observed only for two PPO genes (*PPO1* and *PPO8*) in *Ae*. *albopictus*. In contrast, five PPO genes attained this level of transcript expression (*PPO1*, *PPO2*, *PPO3*, *PPO7* and *PPO9*) in *Cx*. *pipiens* (Fig 5). In general, the magnitude and direction of gene expression was similar in both mosquitoes when infected with either *B*. *bassiana* or *B*. *brongniartii* (Fig 5 and S1 Fig). A few of the exceptions were *PPO1*, *PPO6*, and *PPO7*. For instance, the expression of *PPO1* in *Ae*. *albopictus* was significantly higher (*p* < 0.0001) than controls, irrespective of fungal strain (S1 Fig), while in *Cx*. *pipiens*, only those mosquitoes infected with *B*. *brongniartii* were significantly higher than controls. Furthermore, while *PPO6* expression was significantly enhanced relative to control only in *B*. *bassiana*-infected *Ae*. *albopictus* (*p* = 0.013), there was no effect of fungal infection in *Cx*. *pipiens* (*p* = 0.3475) (Fig 5G–5H).

The main effect of *Wolbachia* infection also differentially affected the expression of PPO genes in these two mosquitoes. The *Wolbachia* effect on expression was significant in one PPO gene (*PPO5*) in *Ae*. *albopictus* (*p* = 0.0437) and three PPO genes (*PPO3*, *PPO5* and *PPO6*) in *Cx*. *pipiens* (*p* = 0.0092, *p* = 0.0442, *p* = 0.0359 respectively) (Table 2). Interestingly, the direction of the *Wolbachia* effect on *PPO5* gene expression differed in *Ae*. *albopictus* and *Cx*. *pipiens*. Here, our bioassays show that while there was a slight but significant increase in *PPO5* expression in *W*^*+*^ *Ae*. *albopictus* compared to their *W*^*-*^ counterparts, a much greater significant decrease in *PPO5* expression occurred in *W*^*+*^ compared to *W*^*-*^ in *Cx*. *pipiens* (Fig 5E and 5F).

### Fungal entomopathogenic infection increases phenoloxidase activity that is dependent on fungal strain and varies with mosquito species

**Fig 6:**
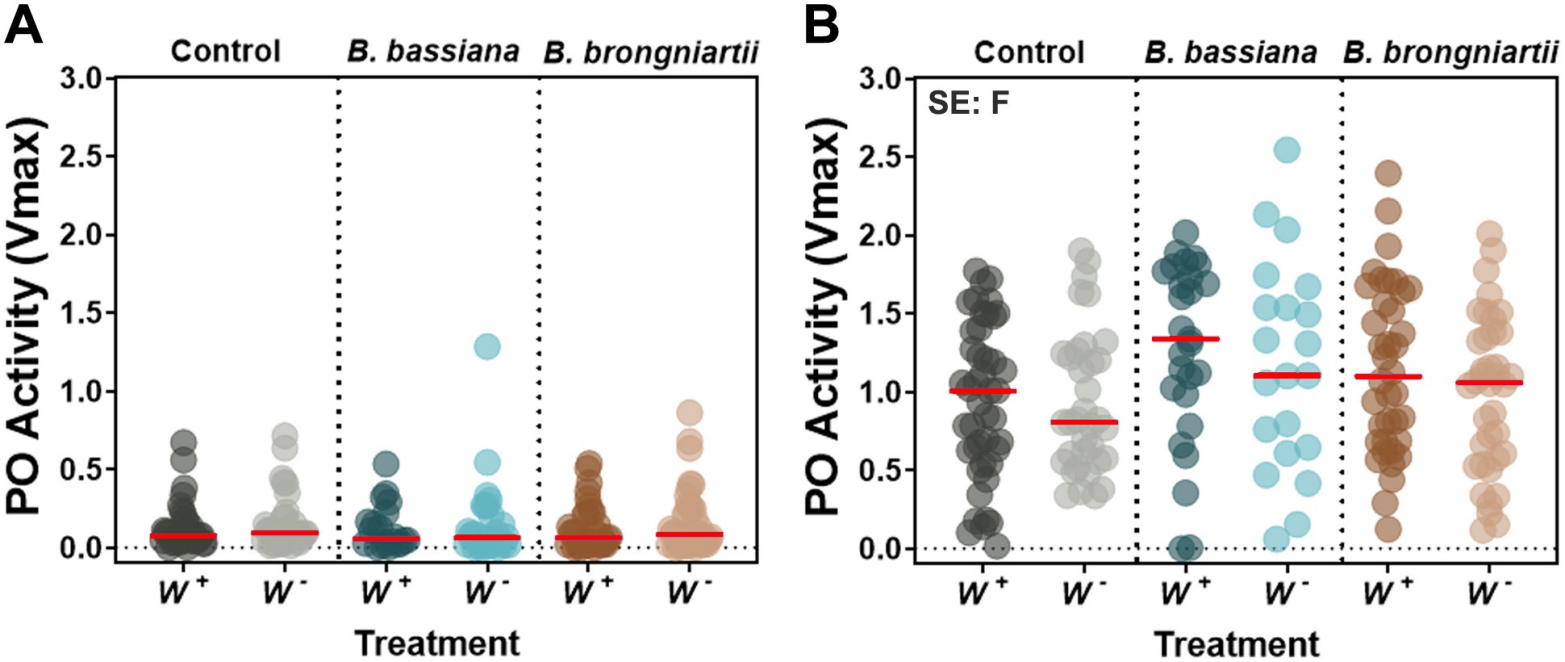
Impact of natural *Wolbachia* and fungal entomopathogenic infections on phenoloxidase activity at 6d post-infection. PO activity in *Ae*. *albopictus* **(A)** and *Cx*. *pipiens* **(B)** mosquitoes. Data represents 21 to 45 samples per treatment originating from three independent experiments. Each dot represents a pool of 2 mosquitoes (*Ae*. *albopictus*) or 1 mosquito (*Cx*. *pipiens*) and the horizontal bar indicates the median level of PO activity. Significant effects (SE) indicate whether the independent factors: Fungal entomopathogen (F), *Wolbachia* presence (W) or their interaction (F*W) were statistically significant. See Table 2 for complete statistics from the Two-Way ANOVA.

To corroborate the PPO gene expression profile observed via qPCR, we also evaluated the whole body phenoloxidase (PO) enzymatic activity in these two mosquitoes under the context of *Wolbachia* and fungal infections. We found no effects of fungal infection, *Wolbachia*, or interaction on PO activity levels in *Ae*. *albopictus*, but a significant, though small, effect of only fungal infection in *Cx*. *pipiens* (*p* = 0.0284) (Table 2). Follow up tests yielded no significant pairwise differences among fungus treatments (Fig 6B). A notable observation was the low levels of PO activity in *Ae*. *albopictus* mosquitoes compared to *Cx*. *pipiens* (Fig 6). Repeated measures with different batches of *Ae*. *albopictus* mosquitoes produced the same results, indicating low basal levels of PO activity in *Ae*. *albopictus* compared to *Cx*. *pipiens* using this method (Fig 6 and S3 Fig).

### Fungal entomopathogenic infection alters the mosquito microbial load

**Fig 7:**
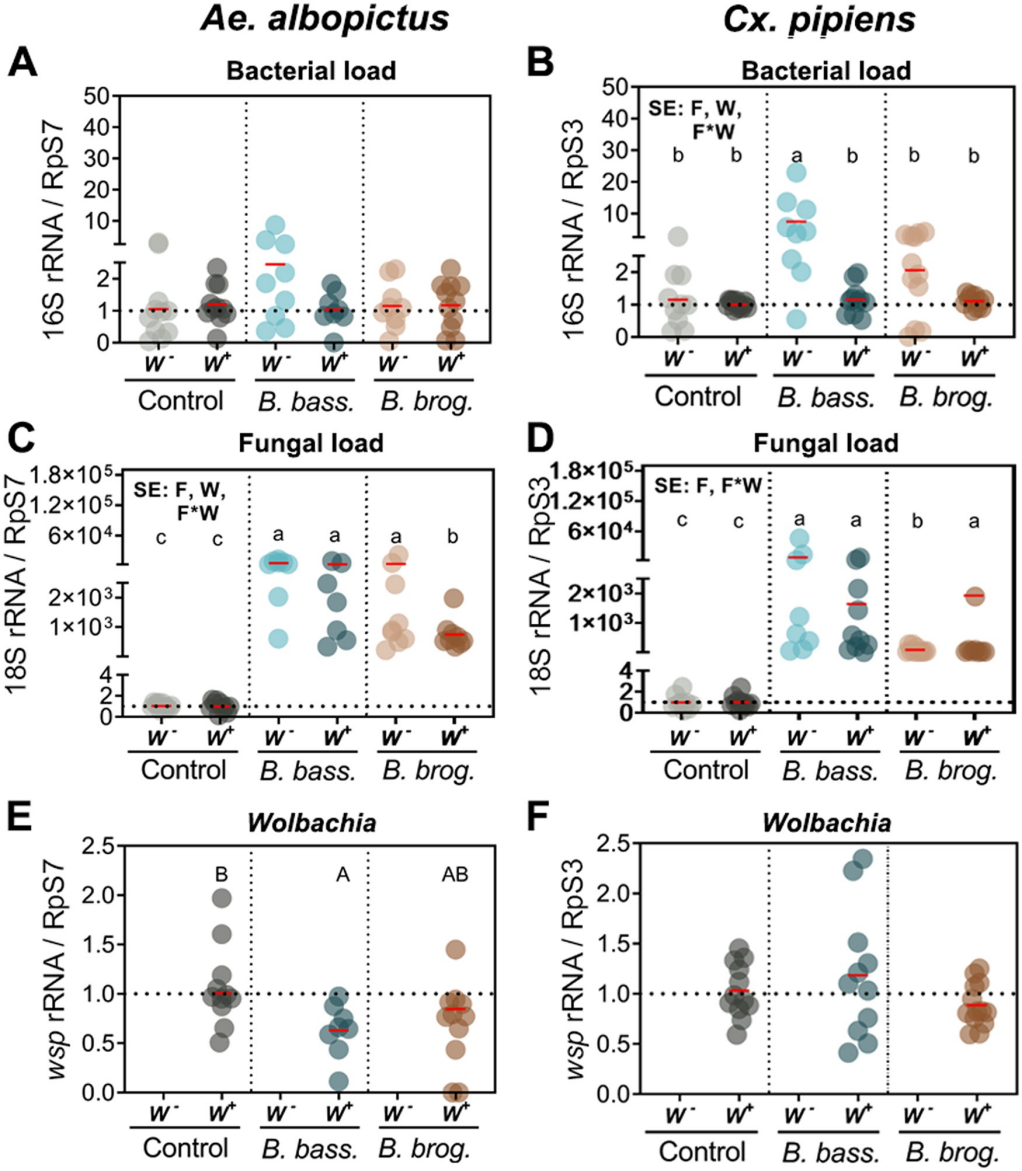
Microbial load following infections with entomopathogenic fungi in mosquitoes with cleared or natural *Wolbachia* infections. Bacterial, fungal and *Wolbachia* loads were measured via the relative quantification of bacterial 16s rRNA (A-B), Fungal 18s rRNA (C-D) and *Wolbachia wsp* (E-F) respectively. Significant effects (SE) indicate whether the independent factors: Fungal entomopathogen (F), *Wolbachia* presence (W) or their interaction (F*W) were statistically significant. Lowercase letters indicate interactive effects (F*W), uppercase letters refer to fungal effects and any *Wolbachia* effect is represented by their mean and standard deviation on the upper right corner of the graph. The red horizontal line indicates LS-means from eight to eleven biological replicates per treatment, originating from at least three independent experiments. Groups sharing the same letter are not significantly different at p<0.05 based on differences of least-squares means. W^-^, *Wolbachia*-free; W^+^, *Wolbachia*-infected; *B*. *bass*., *B*. *bassiana*; *B*. *brog*., *B*. *brongniartii*. See Table 3 for complete statistics from the Two-Way ANOVA. For *Wolbachia* relative abundance means associated with the same letter are not significantly different at p<0.05.

Prior studies have shown that fungal infection leads to an increase in the microbial load of infected mosquitoes [36,43]. Hence, to evaluate any potential effects of *Wolbachia* presence/absence and fungal infection on the proliferation of mosquito bacteria and fungi we conducted a relative quantification of these two microbes via qPCR analysis of 16s rRNA (bacteria) and 18s rRNA (fungi). The analysis revealed no significant main effects or interactions on bacterial load (16s rRNA) in *Ae*. *albopictus* (*p* = 0.3944) but significant effects of *Wolbachia*-fungal infection interaction on bacterial load in *Cx*. *pipiens* (*p* < 0.0001) (Table 3). Bacterial load with *B*. *bassiana* infection in *W*^*-*^ *Cx*. *pipiens* was both significantly greater than corresponding control, and greater than in *B*. *bassiana-*infected *W*^*+*^ *Cx*. *pipiens* (Fig 7B). Bacterial load with *B*. *bassiana* infection in *W*^*+*^ *Cx*. *pipiens* did not differ from corresponding control (Fig 7B). In contrast, infection with *B*. *brongniartii* led to a slight, but not significant, increase in bacterial load of *W*^*-*^ *Cx*. *pipiens*, compared to either corresponding control or to *W*^*+*^ *Cx*. *pipiens* (Fig 7B).

**Table 3:** Interactive effects of 2-way factor ANOVA for microbial load (qPCR Type III Fixed effects). Arrows indicate up or down-regulation of gene expression during fungal or *Wolbachia* infection.

	Target	Effect	*Ae*. *albopictus*			*Cx*. *pipiens*		
			Num/Den DF	F Value	Pr>F	Num/Den DF	F Value	Pr>F
**Microbial** **Load**	**16S** **(Bacteria)**	F	2/53	0.95	0.3944	2/58	**↓**11.52	***<0*.*0001***
		W	1/53	1.14	0.2907	1/58	25.98	***<0*.*0001***
		F*W	2/53	1.88	0.1632	2/58	8.81	***0*.*0005***
	**18S** **(Fungi)**	F	2/47	**↑**587.65	***<0*.*0001***	2/52	**↑**197.03	***<0*.*0001***
		W	1/47	**↓**11.52	***0*.*0014***	1/52	2.05	0.1581
		F*W	2/47	4.63	***0*.*0146***	2/52	15.21	***<0*.*0001***
	***Wolbachia*** **(*wsp*)**	F	2/25	4.06	***0*.*0298***	2/33	2.05	0.1447
		W	-	-	***-***	-	-	-
		F*W	-	-	***-***	-	-	***-***

Fungal loads in both mosquitoes were significantly affected by the interaction of *Wolbachia* and fungal infection (Table 3). In *Ae*. *albopictus*, significantly higher fungal load was observed in all combinations of fungal infection–*Wolbachia* compared to control (*p* = 0.0146). Here, in *B*. *bassiana*-infected *Ae*. *albopictus* there was no difference between *W*^*+*^ *and W*^*-*^ groups, whereas in *B*. *brongniartii*-infected *Ae*. *albopictus* the *W*^*+*^ group had a lower fungal load than did the *W*^*-*^ group (Fig 7C). The *W*^*+*^ *B*. *brongniartii*-infected group also had a significantly lower fungal load than did the *W*^*+*^ *B*. *bassiana*-infected group (Fig 7C). The fungal infection-*Wolbachia* interaction effect was also significant in *Cx*. *pipiens* mosquitoes (*p* < 0.0001), but the direction of the difference in fungal load for *W*^*+*^ vs. *W*^*-*^ groups was opposite of that observed in *Ae*. *albopictus* mosquitoes (compare Fig 7C and 7D).

Finally, we also evaluated the effect of fungal infection on *Wolbachia* relative abundance by measuring the *Wolbachia wsp* gene in these two mosquito populations. Our results indicate a significant effect of fungal infection on *Wolbachia* density for *Ae*. *albopictus* (One-way ANOVA, F_2,25_ = 4.03, p = 0.0304), but not for *Cx*. *pipiens* (One-way ANOVA, F_2,33_ = 2.03, p = 0.1469; Fig 7F). For *Ae*. *albopictus*, there was a slight but significant drop in *Wolbachia* density in mosquitoes infected with *B*. *bassiana* compared to control, but not with *B*. *brongniartii* (Fig 7E). The difference between *Wolbachia* density for *Ae*. *albopictus* infected with the two fungi was also not significant (Fig 7E).

## Discussion

The outcome of entomopathogenic infections is often defined by pathogen virulence, and host genotype. However, other host-associated microbiota can have significant impact on host-pathogen interactions [48–50]. This can be particularly true with naturally occurring symbionts, such as *Wolbachia*, which have evolved to be intimately associated with their insect hosts [51]. Nevertheless, such multipartite interactions during an entomopathogenic infection process are not entirely understood. Thus, in this study, we evaluated the responses of two important mosquito species (*Ae*. *albopictus* and *Cx*. *pipiens*) when challenged with different fungal entomopathogens, with or without native *Wolbachia* infections.

First, we assessed whether natural *Wolbachia* infections could affect mosquito susceptibility to fungal entomopathogens. Our studies comparing *Wolbachia*-infected (W^+^) and *Wolbachia*-free (W^-^) mosquitoes indicate that clearance of *Wolbachia* does not affect mosquito susceptibility to fungal entomopathogens in *Ae*. *albopictus* and *Cx*. *pipiens* mosquitoes. However, it is plausible that different combinations of mosquitoes, *Wolbachia* and fungal entomopathogen genotypes could generate different outcomes. For instance, spider mite (*Tetranychus urticae*) populations naturally infected with *Wolbachia* presented variable effects of fungal infection when challenged with *Metarhizium brunneum* and *B*. *bassiana*. While neither of these fungal entomopathogens had any effect on one spider mite population, *Wolbachia* presence led to an increase in mortality when a different mite population was challenged with *B*. *bassiana* [48]. In addition, a study conducted with the fruit fly *Drosophila melanogaster* reported a protective effect of *Wolbachia* against infection with the entomopathogenic fungus *Beauveria bassiana* [30].

With regards to mosquito susceptibility to fungal entomopathogens, our bioassays suggest that while *Ae*. *albopictus* is susceptible to the strain of *B*. *brongniartii* we used in this assay, *Cx*. *pipiens* mosquitoes appear to be resistant to this fungal entomopathogen, independent of *Wolbachia* infection status.

Next, we evaluated the anti-fungal response repertoire of *Ae*. *albopictus* and *C*. *pipiens* to these two fungal entomopathogens by assessing key immune response markers. These results appear largely similar to that observed in *Ae*. *aegypti* [36,52,53], with fungal entomopathogenic infections presenting itself as a strong independent factor, engaging upstream pathogen recognition receptors such as *PGRP-LC* and *PGRP-S1* and leading to the induction of canonical transcription factors *REL1* and *REL2*, from the Toll and Imd pathway respectively. Previous studies have demonstrated the importance of these two immune pathways in the mosquito defense against entomopathogenic fungi [53–55]. Thus, except for *REL1* in *Cx*. *pipiens* mosquitoes, our results suggest that similar immune signaling pathways are governing the main anti-fungal responses in these two mosquitoes.

Anti-fungal effectors, including antimicrobial peptides and thioester proteins, have been shown to increase in expression in response to entomopathogenic fungal infection [36,53,56]. Our study evaluating orthologs of three *Ae*. *aegypti* antimicrobial peptides (*CECA*, *DEFC* and *LYS-C*), indicates that distinct patterns of expression are occurring in *Ae*. *albopictus* and *Cx*. *pipiens* during fungal and *Wolbachia* infections. For instance, while fungal strain was the only factor significantly increasing *CECA* expression in *Ae*. *albopictus*, *CECA* expression in *Cx*. *pipiens* was significantly affected by both fungal and *Wolbachi*a infections. Our data further suggests that *Wolbachia* is repressing the expression of *CECA* in *Cx*. *pipiens* during an entomopathogenic fungal infection given the significantly higher level of *CECA* expression in *W*^*-*^ compared to its *W*^*+*^ counterpart. In a similar pattern, the expression of defensin appeared to be induced by fungal infection in both mosquito species but in *Cx*. *pipiens* there is a strong interactive effect of fungi and *Wolbachia* affecting *DEFC* expression. This was observed between *W*^*-*^ and *W*^*+*^ *Cx pipiens* mosquitoes, with *W*^*+*^ cohorts exhibiting higher *DEFC* induction. Interestingly, while *B*. *bassiana* induced a higher *DEFC* expression in *Wolbachia*-free *Cx*. *pipiens* mosquitoes, the opposite was true in *W*^*-*^ *Cx*. *pipiens* mosquitoes infected with *B*. *brongniartii*. This might suggest that while *DEFC* is important in the defense against *B*. *bassiana*, it is also employed in the interaction between the *Cx*. *pipiens* mosquito and *Wolbachia*. Alternatively, it might indicate that *B*. *brongniartii* can more efficiently suppress *DEFC* in *Wolbachia*-free *Cx*. *pipiens*. However, the lack of any detrimental effect of *B*. *brongniartii* on the survival of *Cx*. *pipiens* mosquitoes does not lend much support to this possibility. Nevertheless, these diverging patterns of AMP expression might indicate that their elicitation and function is different from its *Ae*. *aegypti* ortholog.

While lysozyme is induced by fungal infection in similar patterns in both mosquitoes, our results indicate that infections by *B*. *bassiana* elicit stronger responses than *B*. *brongniartii*. This phenotype is most likely reflective of the higher replicative nature of *B*. *bassiana* blastospores inside the mosquito during the infection stage, as demonstrated by our fungal load analysis and is comparable to what we observed in *Ae*. *aegypti*-entomopathogenic fungi interactions.

TEP22 is another important anti-fungal effector in *Ae*. *aegypti*, one that is elicited independent of the fungal entomopathogenic strain [36,56]. However, our assays show a divergence from this phenotype, with *TEP22* expression in *Ae*. *albopictus* not affected by fungal infection alone but rather, its induction appears to be regulated by *Wolbachia* presence and dependent on the type of infecting fungal strain. In contrast, *TEP22* expression in *Cx pipiens* is significantly induced with fungal infection, resembling partly what is observed in *Ae*. *aegypti* [36,56]. This most likely indicates that in *Cx*. *pipiens* mosquitoes *TEP22* also functions as an integral part of the anti-fungal repertoire. The slight but significant increase in *Cx*. *pipiens TEP22* expression when *Wolbachia* is absent, compared to present, could indicate that *Wolbachia* is also tightly, and negatively, regulating this important mosquito effector during an infection process, independent of the strain of infecting fungi.

Elicitation of the oxidative pathway and the corresponding antioxidant defense system are crucial components of the mosquito defense against microbial infections [57,58]. Previous studies with *Ae*. *aegypti* have found that fungal infections modulate the state of oxidative stress in the infected mosquito; one that is in turn dependent on the strain of infecting fungi [36]. Interestingly, our study indicates a slight but significant induction of the ROS-generating enzyme DUOX gene in *Ae*. *albopictus* but not in *Cx*. *pipiens* mosquitoes and only with infections with *B*. *bassiana*. This infection-induced ROS production by DUOX is likely reflective of the more virulent characteristics of *B*. *bassiana*, as observed by the higher mosquito mortality associated with *B*. *bassiana* than with *B*. *brongniartii* infection.

To prevent the overstimulation of the oxidative pathway and overgeneration of reactive oxygen species, a set of detoxifying enzymes are set in place to regulate this process and avoid cellular damage [58–60]. Our gene expression analysis of antioxidant defense genes indicate that the catalase gene is a critical component in the mosquito responses to fungal infections. However, the responses are drastically different in *Ae*. *albopictus* and *Cx pipiens* mosquitoes. The interactive effect of fungi and *Wolbachia* infection in *Ae albopictus* mosquitoes appear to suggest that while fungal infection by itself does not affect catalase elicitation, *Wolbachia* could be dampening catalase induction only under infections with a less pathogenic fungi such as *B*. *brongniartii*, which does not appear to induce *DUOX*. Alternatively, *B*. *brongniartii* could be eliciting other ROS-generating enzymes not evaluated in our study, which in turn might be inducing catalase expression when the mosquito is free from *Wolbachia* regulation in *W*^*-*^ *Ae*. *albopictus* mosquitoes. In contrast, *Cx pipiens* display a diverging phenotype, with significant catalase elicitation only under the presence of *Wolbachia* and with stronger induction during infections with the less virulent *B*. *brongniartii* entomopathogenic fungi. Whether the high catalase induction observed in *Cx pipiens* mosquitoes is linked to the mosquito resistant phenotype against *B*. *brongniartii* infection remains to be elucidated; but our results indicate that *Wolbachia* is playing a dynamic role in the mosquito antioxidant responses to infections by fungal entomopathogens. In this context, while *Wolbachia* interactions with oxidative stress have been documented in transfected hosts, our data suggests that native *Wolbachia* might be involved in maintaining host redox homeostasis during a pathogenic infection process, as previously hypothesized [60].

The prophenoloxidase cascade is another important anti-fungal response mechanism that has been observed in several insects [37,53,61]. In *Ae*. *aegypti*, its expression is affected by fungal pathogenic strain and by the progression of fungal infection, with higher PPO gene expression observed at the later stages of infection [36,37]. In our assays, the absence of any interactive effect of *Wolbachia* presence and fungal entomopathogenic infection in *Ae*. *albopictus* mosquitoes demonstrates that these genes are tightly linked to either the anti-fungal response or *Wolbachia* symbiotic homeostasis. In contrast, our bioassays with *Cx pipiens* indicated a highly significant interactive effect of fungi and *Wolbachia* infection on the expression of one PPO gene (*PPO2*). In this interaction, while fungal infection elicited *PPO2* expression, infections by the most lethal fungi *B*. *bassiana* failed to induce *PPO2* when *Wolbachia* was present. Given the absence of this phenotype with *B*. *brongniartii*, our data might suggest that under the physiological conditions provided by the more virulent entomopathogenic fungus, *Wolbachia* is able to limit the action of a potent *PPO2* and avoid damage to its host cell. Whether the same phenotype occurs with an *Ae*. *albopictus* PPO gene that we did not test remains to be seen, but our efforts to locate a *PPO2* ortholog in *Ae*. *albopictus* were not successful.

Furthermore, the PPO cascade gene members in these two mosquito species show distinct expression profiles, potentially indicating that they are playing diverging roles in the response to fungal infection and *Wolbachia* homeostasis. For instance, while two PPO genes (*PPO1* and *PPO8*) show the highest degree of gene expression in response to fungal infection in *Ae*. *albopictus*, five PPO genes showed higher transcript abundance in *Cx*. *pipiens* (*PPO1*, *PPO2*, *PPO5*, *PPO7* and *PPO9*). Whether these genes are playing the same role in these two mosquitoes remains to be elucidated, but their expression profile might explain why *Cx*. *pipiens* is less susceptible to *B*. *bassiana* and especially resistant to infection by the fungal entomopathogen *B*. *brongniartii*. Furthermore, our assays conducted to corroborate our PO gene expression profiles, partly support this finding, with an increase in PO activity in *Cx*. *pipiens* mosquitoes infected with *B*. *bassiana* and no increase in PO activity levels with *B*. *brongniartii*. These results differ from the significant drop of PO activity that has been observed in *Ae*. *aegypti* when challenged with a range of fungal entomopathogenic strains [36,37]. While pre-infection levels of PO activity in *Cx*. *pipiens* could potentially determine this phenotype, our analysis on the basal levels of PO activity shows similar profiles between *Ae*. *aegypti* and *Cx pipiens*, indicating that the observable values are a true representation of their diverging responses to the same entomopathogenic fungal strains. Alternatively, it could also mean that *Cx pipiens* are much more resistant to the potential immune suppressive activity of these entomopathogenic fungi.

While our PO activity analysis in *Ae*. *albopictus* was inconclusive, our transcript abundance analysis indicates a dynamic gene expression for some PO cascade gene members. Repeated measures with different batches of *Ae*. *albopictus* mosquitoes produced the same results, potentially suggesting that *Ae*. *albopictus* maintains a low basal PO activity level, one that is maintained independent of fungal infection. Alternatively, our failure to successfully measure PO enzymatic activity in *Ae*. *albopictus*, could indicate an *Ae*. *albopictus*-derived inhibitor affecting our methodology rather than an absence of PO activity in this mosquito.

Our study also shows a potential *Wolbachia* interaction with its native host at the PPO cascade, given the *Wolbachia* modulation of two PPO genes in each mosquito species. This might suggest that some of these PO genes are involved in *Wolbachia* maintenance. In a study that included *Drosophila melanogaster*, *D*. *simulans* and *Ae*. *aegypti*, Thomas et al. [62] demonstrated an increase in melanization in all three dipterans infected with *Wolbachia wMelPop*; indicating an interaction between this symbiont and the insect melanization cascade. Further studies evaluating the role that the prophenoloxidase cascade plays in the maintenance of natural *Wolbachia* infections could add to our understanding of the tightly woven interaction between this endosymbiotic microbe and its mosquito host. Although, some studies have found no impact of *Wolbachia* on insect immunity [63,64], these studies did not evaluate the effects of *Wolbachia* under the context of an active coinfection with a microbial pathogen. For instance, Blagrove et al [65] showed no significant immune regulation when an *Ae*. *albopictus Wolbachia*-free line was transiently infected with *w*MelPop or wAlbB strains, or with heat-killed *Escherichia coli*. Blagrove et al [65] suggest that the absence of a robust immune induction during *Wolbachia* transinfection might be due to *Ae*. *albopictus* immunotolerance to *Wolbachia*. In turn, the lack of any significant immune induction with *E*. *coli* might be due to the nature of this microbe (heat-killed) not providing the same level of immune challenge of an actively replicating microbe. Thus, it is plausible that such impacts on immunity are much more apparent under stress (i.e., another infection) as has been previously suggested [60,66].

Finally, we evaluated the effects of entomopathogenic fungal infection and *Wolbachia* on components of the microbial community of these mosquitoes, given that prior studies have shown significant alterations on bacterial load during fungal entomopathogenic infections [36,43]. Our analysis revealed a significant interactive effect of *Wolbachia* presence and fungal entomopathogenic strain on bacterial load in *Cx*. *pipiens* mosquitoes but its absence in *Ae*. *albopictus*. This contrasting interactive effect on bacterial load might reflect the specific mosquito immune responses mounted against the different fungal strains and the *Wolbachia* strain-specific interaction with its host. Our data suggests that these responses might be driven by AMPs, especially by *CECA* and *DEFC*, given the significant effect of *Wolbachia* on *CECA* expression and the interactive effect of fungi-*Wolbachia* on *DEFC* expression observed in *Cx*. *pipiens* mosquitoes. In addition, it is possible that other antimicrobial peptides or genes governing gut microbiota homeostasis in mosquitoes are also being affected by this interaction. A plausible alternative explanation could be that the differences in bacterial load are due to the removal of native bacteria following tetracycline treatment of the parent pool. Given that this study only assessed bacterial load and not bacterial diversity, we are unable to determine with certainty if this is the case. However, other than *Wolbachia*, there is no other bacterium that is known to be transmitted vertically in *Ae*. *albopictus* and *Cx*. *pipiens* mosquitoes. The symbiotic bacterium *Asaia spp*. has been shown to infect different tissues and be present on egg surfaces of other mosquito species that do not harbor *Wolbachia* (*Anopheles spp*, and *Aedes aegypti*) but has not been found on eggs of *Ae*. *albopictus* or *Cx*. *pipiens* [67]. Furthermore, as part of the transition from larvae to adult, and in a process that involves microbiota encapsulation and excretion in the meconium, there is almost a complete removal of midgut bacteria in newly emerged adults [68]. Thus, it is not surprising that most of the mosquito core gut microbiota are acquired from the environment (larval habitat or from a sugar/plant source as adults) [69–71].

Our studies also show diverging results when we measured the total fungal load in these mosquitoes, potentially indicating that the infection-derived responses and fungal-*Wolbachia* interactions were having different effects on these two mosquito species. However, these different phenotypes were only observed under the context of *B*. *brongniartii* infections and suggests that *B*. *bassiana* proliferation are not affected by the presence of *Wolbachia* in any of the two mosquito species. Our study also indicates that entomopathogenic fungal infection has a detrimental effect on *Wolbachia* density, one that is dependent on fungal strain and mosquito host. This drop in *Wolbachia* loads in *B*. *bassiana*-infected *Ae*. *albopictus* mosquitoes might reflect the higher toxicity of this fungus and/or that many more tissues are compromised in this mosquito during *B*. *bassiana* infections. This might be supported by our data if we consider the earlier mortality observed in *Ae*. *albopictus* compared to *Cx*. *pipiens* during infections with *B*. *bassiana*.

In summary, our study shows complex interactions involving entomopathogenic fungal infections under the context of native *Wolbachia* infections. While some of the anti-fungal host responses from *Ae*. *albopictus* and *Cx*. *pipiens* are similar, there are distinct differences with regards to the direction and magnitude of expression observed post-fungal infection. This was true for gene members of important mosquito immune functions such as canonical signaling pathways, AMPs, oxidative/detoxification genes, and the PO cascade, known critical components of the mosquito’s anti-fungal repertoire. One potential limitation of our study is that it did not assess any potential genetic variation that might exists between *Wolbachia*-infected and its *Wolbachia*-free counterparts. It is plausible that slight genetic changes (i.e via genetic drift) may have occurred during the tetracycline treatment that could affect the interpretation of our results. Although, the *Wolbachia*-fungi interactive effects we observed does not appear to impact mosquito survival to entomopathogenic infections, they might influence other important vector biology parameters such as vector competence/capacity and mosquito reproduction. To our knowledge this is the first study to evaluate fungal entomopathogenic infections under the context of a native mosquito symbiont. Given the inclusion of *Wolbachia* in alternative methods of mosquito and mosquito-borne pathogen control, this study provides a snapshot of the mosquito susceptibility and immune responses when challenged with fungal entomopathogens and under the context of native *Wolbachia* infections.

## Supporting information

S1 Table Primer sequences used in qPCR. (XLSX)

S2 Table Interactive effects of 2-way ANOVA for PO activity (Vmax) (Type III Fixed effects). (XLSX)

S1 Text Phylogenetic trees of select immune gene targets. Trees were built in VectorBase with the resulting protein gene sequences from their respective OrthoMCL’s ortholog groups.
(DOCX)

S1 Fig Expression of oxidative stress or the antioxidant response genes during fungal entomopathogenic infection and under the context of native *Wolbachia* infections. Significant effects (SE) indicate whether the independent factors: Fungal entomopathogen (F), *Wolbachia* presence (W) or their interaction (F*W) were statistically significant. The red horizontal line indicates LS-means. Uppercase letters refer to fungal effects and groups sharing the same letter are not significantly different at p<0.05 based on differences of least-squares means. W^-^, *Wolbachia*-free; W^+^, *Wolbachia*-infected; *B*. *bass*., *B*. *bassiana*; *B*. *brog*., *B*. *brongniartii*. See Table 3 for complete statistics from the Two-Way ANOVA.
(TIF)

S2 Fig Gene expression of oxidative stress and detoxification genes during natural *Wolbachia* and fungal entomopathogenic infections. Significant effects (SE) indicate whether the independent factors: Fungal entomopathogen (F), *Wolbachia* presence (W) or their interaction (F*W) were statistically significant. The red horizontal line indicates LS-means. Uppercase letters refer to fungal effects and groups sharing the same letter are not significantly different at p<0.05 based on differences of least-squares means. W^-^, *Wolbachia*-free; W^+^, *Wolbachia*-infected; *B*. *bass*., *B*. *bassiana*; *B*. *brog*., *B*. *brongniartii*. See S2 Table for complete statistics from the Two-Way ANOVA.
(TIF)

S3 Fig Basal levels of phenoloxidase activity in *Ae*. *albopictus* and *Cx*. *pipiens* mosquitoes. Data analyzed via single-factor ANOVA using PROC GLIMMIX with a gamma distribution in SAS. Species mean Vmax rates sharing the same letter are not significantly different at p<.05 based on differences of least-squares means.
(TIF)
