# Supplementary material for: Impacts of fungal entomopathogens on survival and immune responses of Aedes albopictus and Culex pipiens mosquitoes in the context of native Wolbachia infections
Source: PLoS Negl Trop Dis. 2021 Nov 29;15(11):e0009984. doi: 10.1371/journal.pntd.0009984 (PMC8670716; doi:10.1371/journal.pntd.0009984)

**S1 Text.- Phylogenetic trees of select immune gene targets.** Trees were built in VectorBase with the resulting protein gene sequences from their respective OrthoMCL’s ortholog groups.


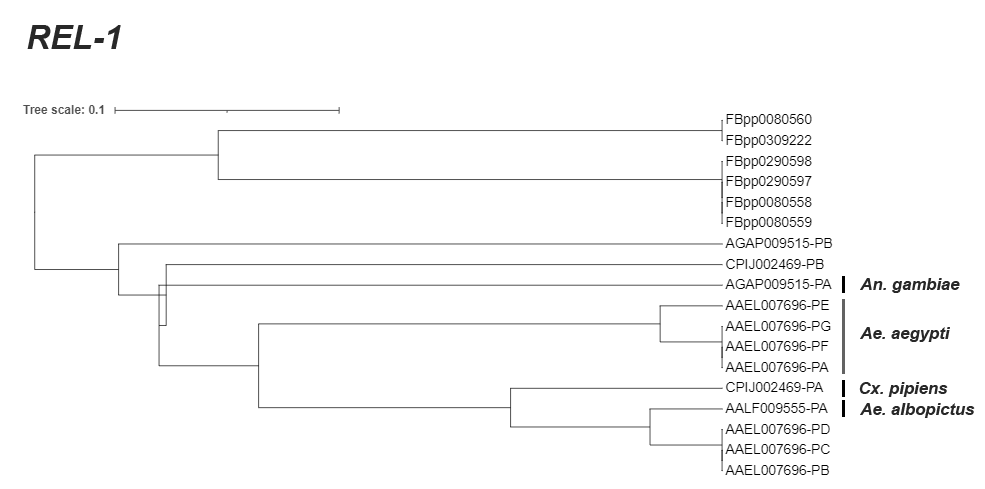


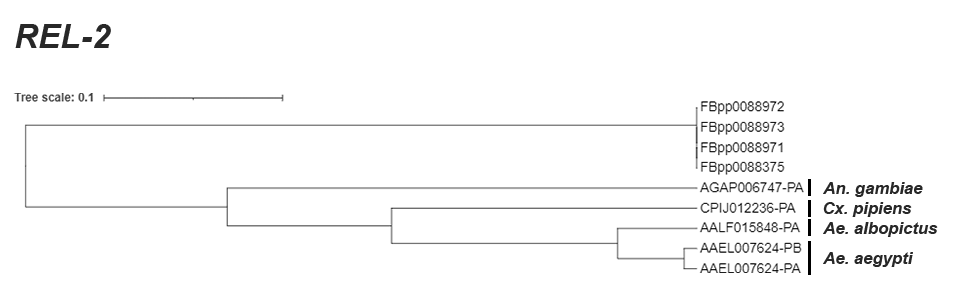


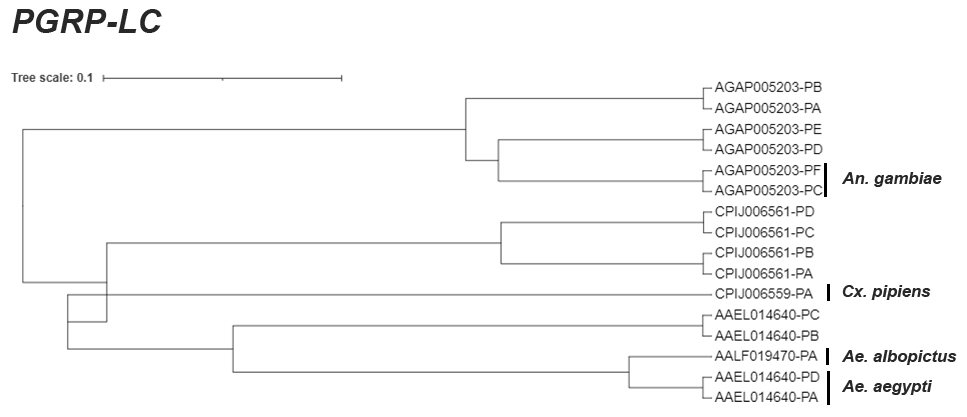


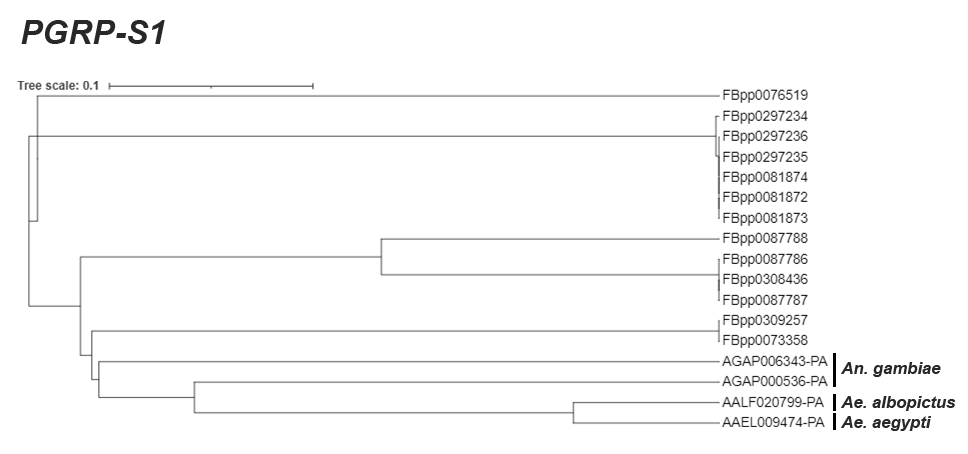


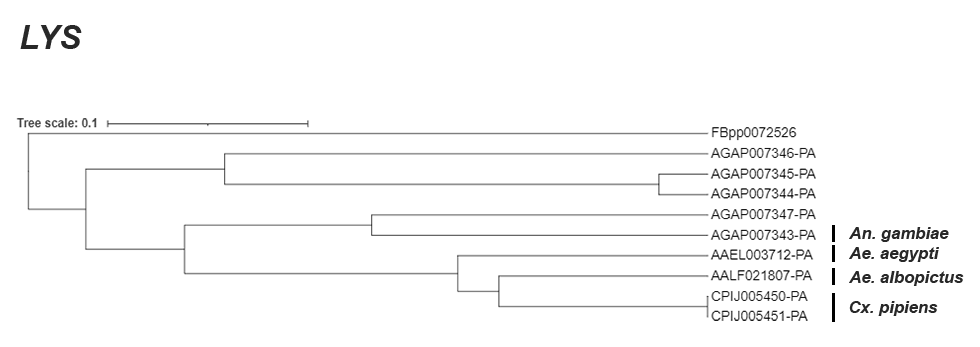


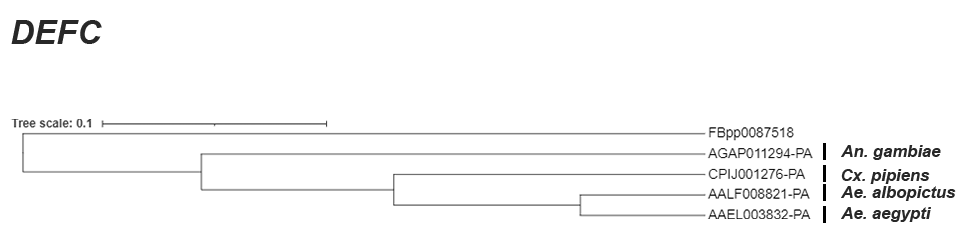


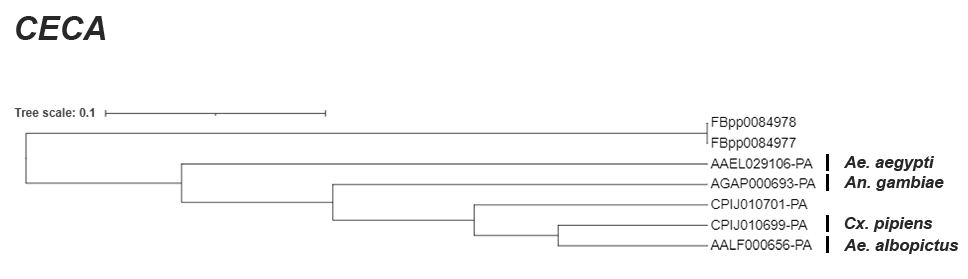


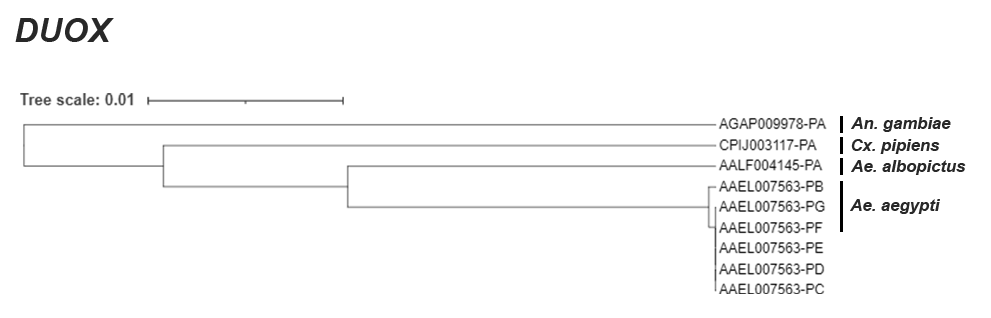


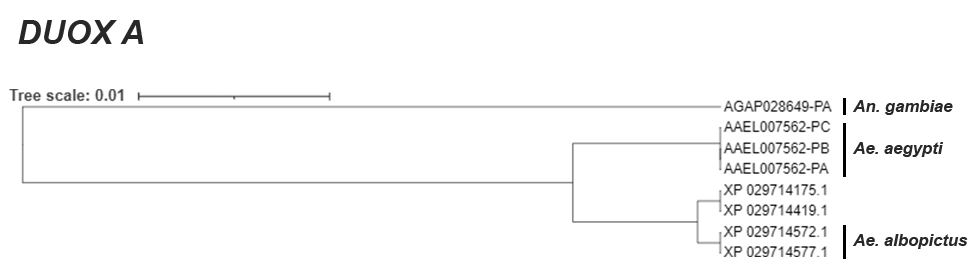


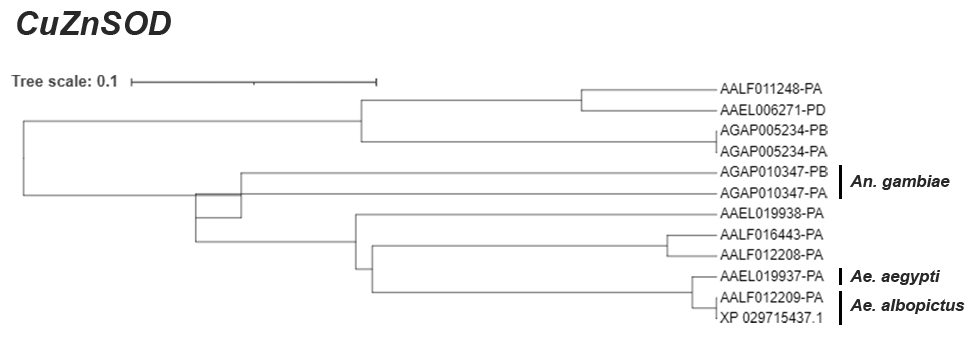


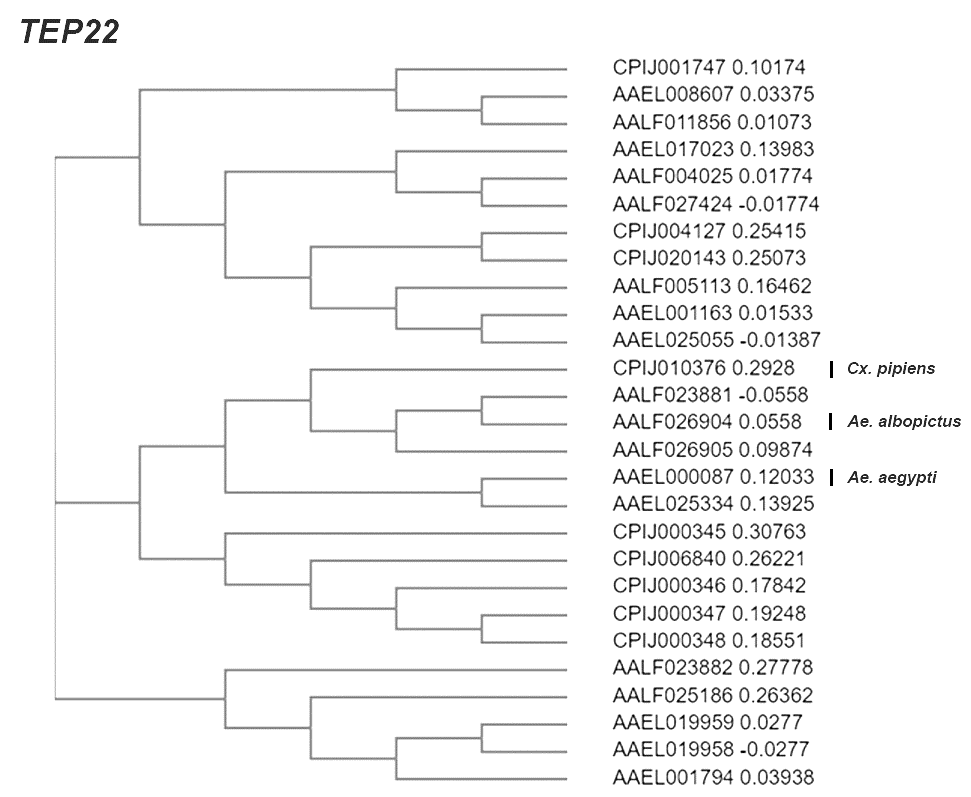


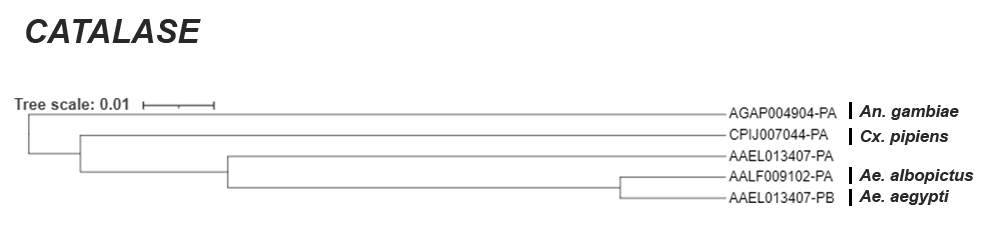


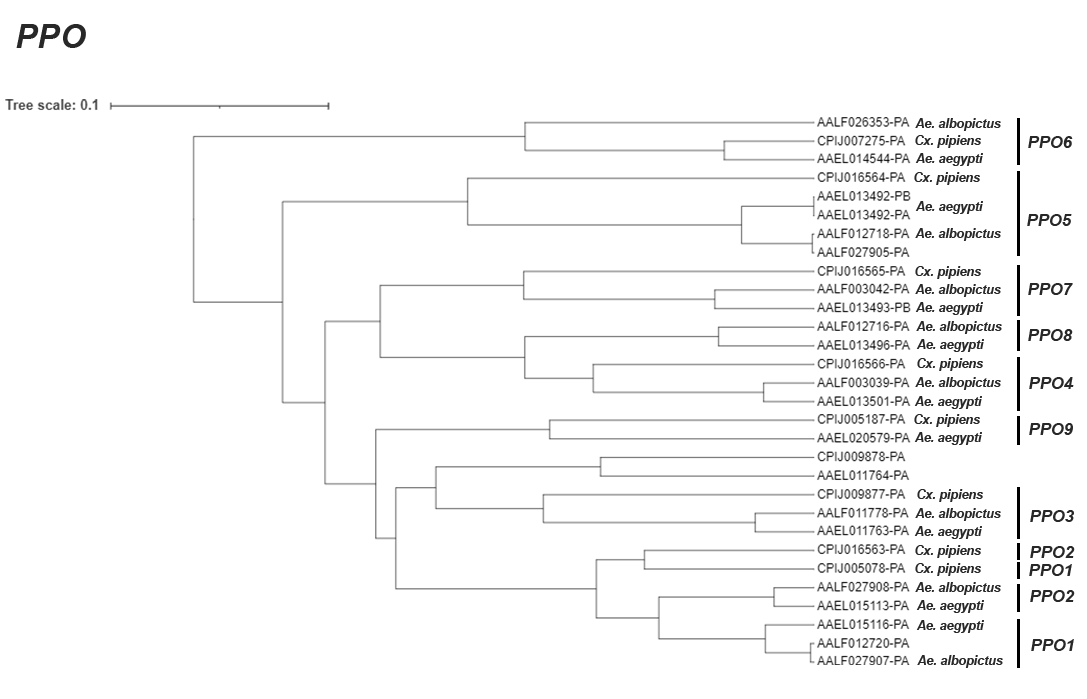

Supplement: S1 Text — Trees were built in VectorBase with the resulting protein gene sequences from their respective OrthoMCL’s ortholog groups. (DOCX) [file pntd.0009984.s003.docx]
