# Supplementary figures and images for: Impacts of fungal entomopathogens on survival and immune responses of Aedes albopictus and Culex pipiens mosquitoes in the context of native Wolbachia infections
Source: PLoS Negl Trop Dis. 2021 Nov 29;15(11):e0009984. doi: 10.1371/journal.pntd.0009984 (PMC8670716; doi:10.1371/journal.pntd.0009984)

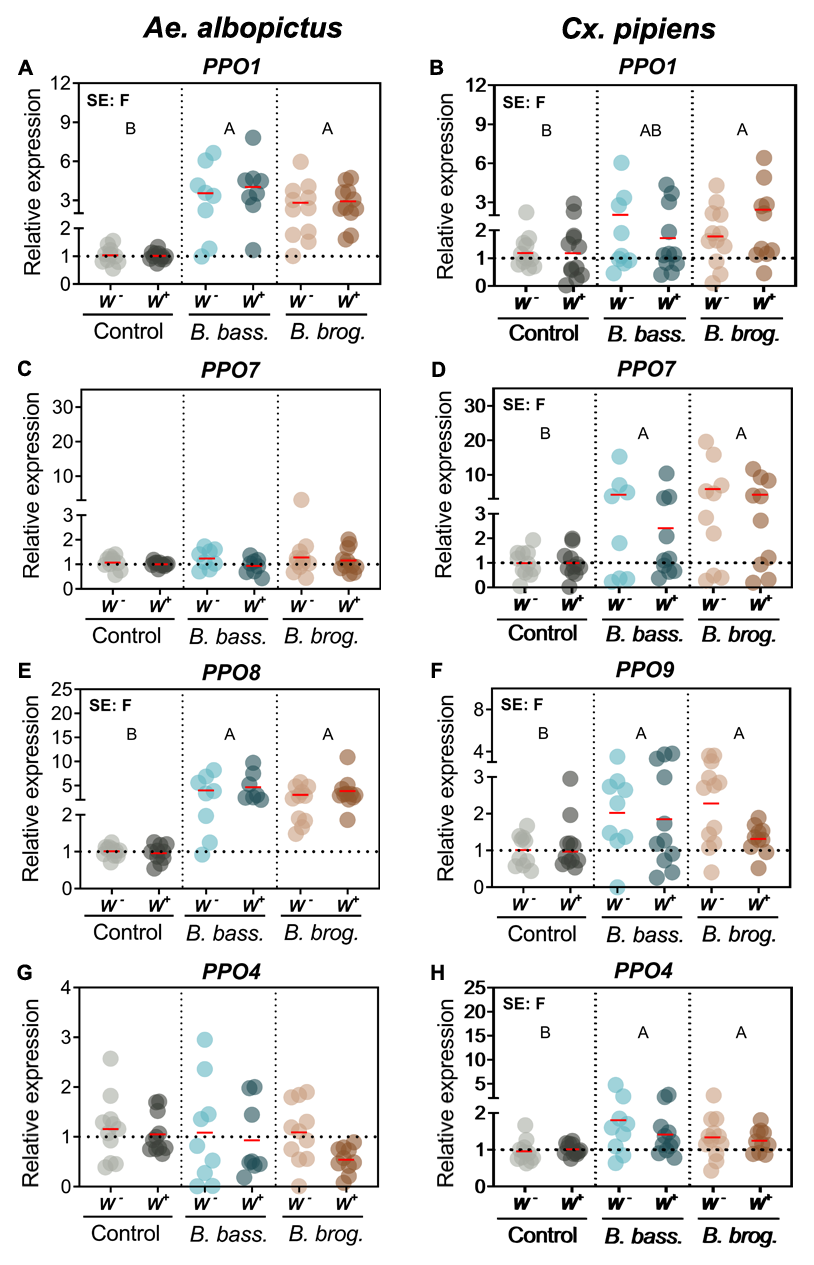

Supplement: S1 Fig — Significant effects (SE) indicate whether the independent factors: Fungal entomopathogen (F), Wolbachia presence (W) or their interaction (F*W) were statistically significant. The red horizontal line indicates LS-means. Uppercase letters refer to fungal effects and groups sharing the same letter are not significantly different at p<0.05 based on differences of least-squares means. W-, Wolbachia-free; W+, Wolbachia-infected; B. bass., B. bassiana; B. brog., B. brongniartii. See Table 3 for complete statistics from the Two-Way ANOVA. (TIF) [file pntd.0009984.s004.tif]

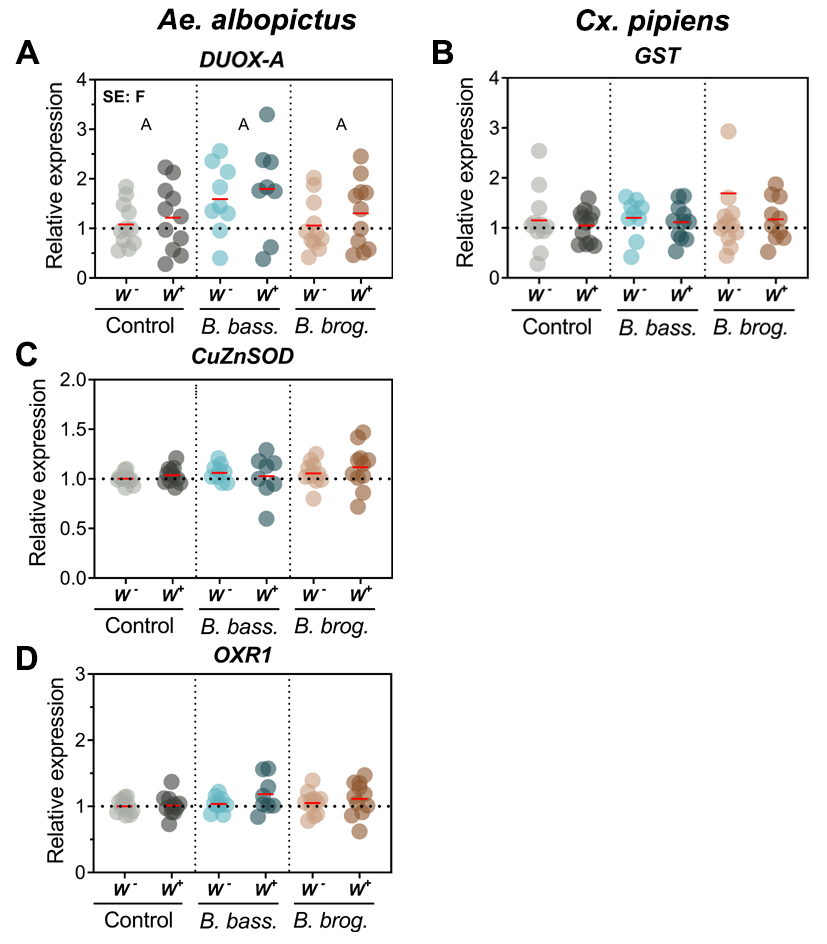

Supplement: S2 Fig — Significant effects (SE) indicate whether the independent factors: Fungal entomopathogen (F), Wolbachia presence (W) or their interaction (F*W) were statistically significant. The red horizontal line indicates LS-means. Uppercase letters refer to fungal effects and groups sharing the same letter are not significantly different at p<0.05 based on differences of least-squares means. W-, Wolbachia-free; W+, Wolbachia-infected; B. bass., B. bassiana; B. brog., B. brongniartii. See S2 Table for complete statistics from the Two-Way ANOVA. (TIF) [file pntd.0009984.s005.tif]

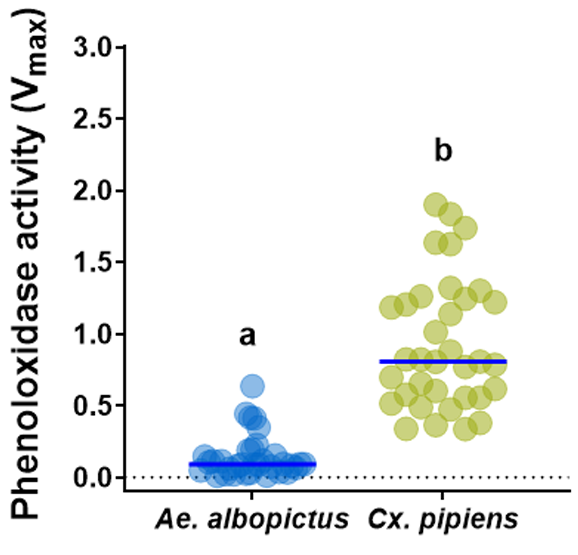

Supplement: S3 Fig — Data analyzed via single-factor ANOVA using PROC GLIMMIX with a gamma distribution in SAS. Species mean Vmax rates sharing the same letter are not significantly different at p<.05 based on differences of least-squares means. (TIF) [file pntd.0009984.s006.tif]
